# Incorporating stakeholders’ preferences into a multi-criteria framework for planning large-scale Nature-Based Solutions

**DOI:** 10.1007/s13280-020-01419-4

**Published:** 2020-12-01

**Authors:** Laddaporn Ruangpan, Zoran Vojinovic, Jasna Plavšić, Dong-Jiing Doong, Tobias Bahlmann, Alida Alves, Leng-Hsuan Tseng, Anja Randelović, Andrijana Todorović, Zvonimir Kocic, Vladimir Beljinac, Meng-Hsuan Wu, Wei-Cheng Lo, Blanca Perez-Lapeña, Mário J. Franca

**Affiliations:** 1 Department of Water Resources and Ecosystems, IHE Delft Institute for Water Education, Westvest 7, 2611 AX Delft, The Netherlands; 2 Department of Hydraulic Engineering, Faculty of Civil Engineering and Geosciences, Delft University of Technology, Stevinweg 1, 2628 CN Delft, The Netherlands; 3 Department of Water Supply, Sanitation and Environmental Engineering, IHE Delft Institute for Water Education, Westvest 7, 2611 AX Delft, The Netherlands; 4 Faculty of Civil Engineering, University of Belgrade, PO Box 42, 11120 Belgrade, Serbia; 5 National Cheng Kung University, 1, University Road, Tainan, 70101 Taiwan; 6 College of Engineering, Mathematics and Physics, University of Exeter, Exeter, UK; 7 Avans University of Applied Sciences, Onderwijsboulevard 215, 5223 DE Hertogenbosch, The Netherlands; 8 Srbijavode, Bulevar umetnosti 2a, 11070 Belgrade, Serbia

**Keywords:** Climate change mitigation, Flood risk reduction, Multi-criteria analysis, Nature-Based Solutions, River basin

## Abstract

Hydro-meteorological risks are a growing issue for societies, economies and environments around the world. An effective, sustainable response to such risks and their future uncertainty requires a paradigm shift in our research and practical efforts. In this respect, Nature-Based Solutions (NBSs) offer the potential to achieve a more effective and flexible response to hydro-meteorological risks while also enhancing human well-being and biodiversity. The present paper describes a new methodology that incorporates stakeholders’ preferences into a multi-criteria analysis framework, as part of a tool for selecting risk mitigation measures. The methodology has been applied to Tamnava river basin in Serbia and Nangang river basin in Taiwan within the EC-funded RECONECT project. The results highlight the importance of involving stakeholders in the early stages of projects in order to achieve successful implementation of NBSs. The methodology can assist decision-makers in formulating desirable benefits and co-benefits and can enable a systematic and transparent NBSs planning process.

**Electronic supplementary material:**

The online version of this article (10.1007/s13280-020-01419-4) contains supplementary material, which is available to authorized users.

## Introduction

Hydro-meteorological risks, such as flooding, will become more extreme and increase in frequency in the foreseeable future. These risks are identified as one of the most likely and impacting risks in global reports (World Economic Forum 2019), as they cause a significant impact on human life, the economy and the environment. After a heavy rain or other extreme weather events, various types of inundation can occur, such as flash floods in steep areas, fluvial floods in floodplains, pluvial floods in urban areas and storm surges in coastal zones (WMO 2011). According to EM-DAT (2017), between 1951 and 2017, floods caused US$ 765 billion of damage and killed almost 24 million people globally. These statistics show that there is an urgent need to develop effective flood management and mitigation measures to minimise consequences as much as possible.

In the past, the most common approaches to reduce flood risks were related to ‘hard’ engineering works or the so-called grey infrastructure (EEA 2017). Examples of such measures include construction of dams, dikes, levees, pipe systems and other structures to control flooding. Generally, grey infrastructure solely reduces hazards in the considered areas, but does not necessarily bring additional benefits, nor does it deal with the future uncertainties related to climate change, land use change and urbanisation. Past experiences with risk strategies have clearly shown that implementing grey infrastructure alone cannot provide complete protection (EEA 2017), due to its inability to adequately adapt to future uncertainty and increasing climate change (Courtney et al. 2013; UNEP 2014). Furthermore, grey infrastructure often has negative consequences in the environment and ecosystems.

The concept of Nature-Based Solutions (NBSs) has been used to describe measures that can be used for both hydro-meteorological risk reduction and climate change adaptation and mitigation while at the same time enhancing ecosystems (e.g. Debele et al. 2019). The term NBS is often used as an umbrella term for many concepts such as Low Impact Developments (LIDs), Best Management Practices (BMPs), Water Sensitive Urban Design (WSUD), Sustainable Urban Drainage Systems (SuDS), Green Infrastructure (GI), Blue-Green Infrastructure (BGI), Ecosystem-based Adaptation (EbA) and Ecosystem-based Disaster Risk Reduction (Eco-DRR). These terms are mainly used to address small-scale NBSs which are applied at the urban or local scale, whereas large-scale NBSs are usually applied in rural areas, river basins and/or at the regional scale (Ruangpan et al. 2020).

However, selecting appropriate NBS measures is still a challenge due to specific local constraints and social-economic conditions (Ruangpan et al. 2020). No single NBS can solve all problems and NBSs are not yet easy to implement in practice. The most suitable solution will depend on local necessities and characteristics. To improve acceptance and implementation of NBSs, decision support tools can be used by considering multiple stakeholders’ views, trade-offs and feasible measures (De Brito and Evers 2016). A flexible decision tool capable of integrating multiple objectives is thus required.

The methods and tools facilitating selection of appropriate NBS measures are reviewed by Jayasooriya and Ng (2014), Lerer et al. (2015), Alves et al. (2018b) and Ruangpan et al. (2020). Most previous studies only focus on urban areas and are still far from being able to systematically support integrated assessment of NBS. Multi-Criteria Analysis (MCA), or as it is sometimes called Multi-Criteria Decision-Making (MCDM), is one of the most popular decision support tools in hydro-meteorological risk management. It can provide a systematic framework to deal with complex decision-making situations with multiple objectives.

There is an extensive literature on MCA application in flood risk management that has been reviewed by De Brito and Evers (2016). MCA techniques have been employed in a wide variety of flood risk problems, namely, Shivaprasad Sharma et al. (2018) for flood risk assessment; Dang et al. (2011) for evaluation of the most important flood risk parameters; Fernández and Lutz (2010) for flood hazard mapping, Azibi and Vanderpooten (2003) for selecting grey infrastructures to reduce flood risk and Shan et al. (2012) for reservoir flood control and emergency management problems. However, few applications of MCA tools exist for the selection of NBS measures.

Martin et al. (2007) carried out the first application of MCA for LID/BMP selection by applying Elimination and Choice that Translates Reality (ELECTRE) for the analysis. Young et al. (2010), Aceves and Fuamba (2016) and Alves et al. (2018b) used stakeholder weighting for criteria such as water quality, environmental and economic benefits, but not for the measures. The stakeholders’ weighting of measures is important, since it can be used to enhance identification of the suitable measures for the specific case study. Loc et al. (2017) collected stakeholders’ NBS preferences, but these preferences were not included in the MCA. From the studies referenced above, it can be seen that there are still some barriers in applying MCA for NBS: (i) they have only been applied to pluvial floods at the urban scale; (ii) weighting for measures are not included in MCA process and (iii) only a few co-benefits have been included as criteria in MCA.

Given these knowledge gaps, this study aims to develop a methodology for the first time to select NBS measures by integrating a preliminary selection tool with a multi-criteria analysis framework for different scales (i.e. urban area, river basin, coastal area) and hazard types (i.e. pluvial floods, fluvial floods, flash flood, coastal floods drought and landslides). This new methodology also incorporates stakeholders’ preferences for both assessment criteria and potential measures into the MCA framework. Involving stakeholders into an MCA can introduce additional relevant local data and considerations into the process of measure selection that might otherwise be unnoticed/disregarded by the engineers. In this way, a selection of the most suitable and effective measures for a specific area and hazard type is ensured. This is important for the successful implementation and sustainable exploitation of a specific measure and, therefore, for long-term risk reduction and effective water resources management. Another highlight of this methodology is that it includes a wide range of criteria for both main benefit (reduction of hydro-meteorological risks) and co-benefits (improvement of water quantity, protection and enhancement of habitats, safeguard of biodiversity and socio-economic and human well-being).

For proof of concept, the proposed methodology has been used in the planning of NBS measures to reduce the impact of fluvial flooding at the river basin scale. NBS measures have been selected and ranked for two case studies within the EC-funded RECONECT project, namely, the Tamnava River basin in Serbia and the Nangang River in Taiwan.

## Methodology for selecting measures

### Methodology structure

This section describes the overall methodology used for selecting potential measures, as well as the database of NBS used as an input. To set up the database, a large set of measures for hydro-meteorological risk reduction has been collected based on the literature review of adaptation and mitigation measures, including grey infrastructure, river restoration, NBSs and their related terms (i.e. LIDs, BMPs, WSUD, SuDS, GI, BGI, EbA, Eco-DRR). The collected information for each measure includes its description, spatial scale of applicability (e.g. river basin, urban area and coastal zone), possible locations for implementation, properties, and possible benefits.

**Fig. 1:**
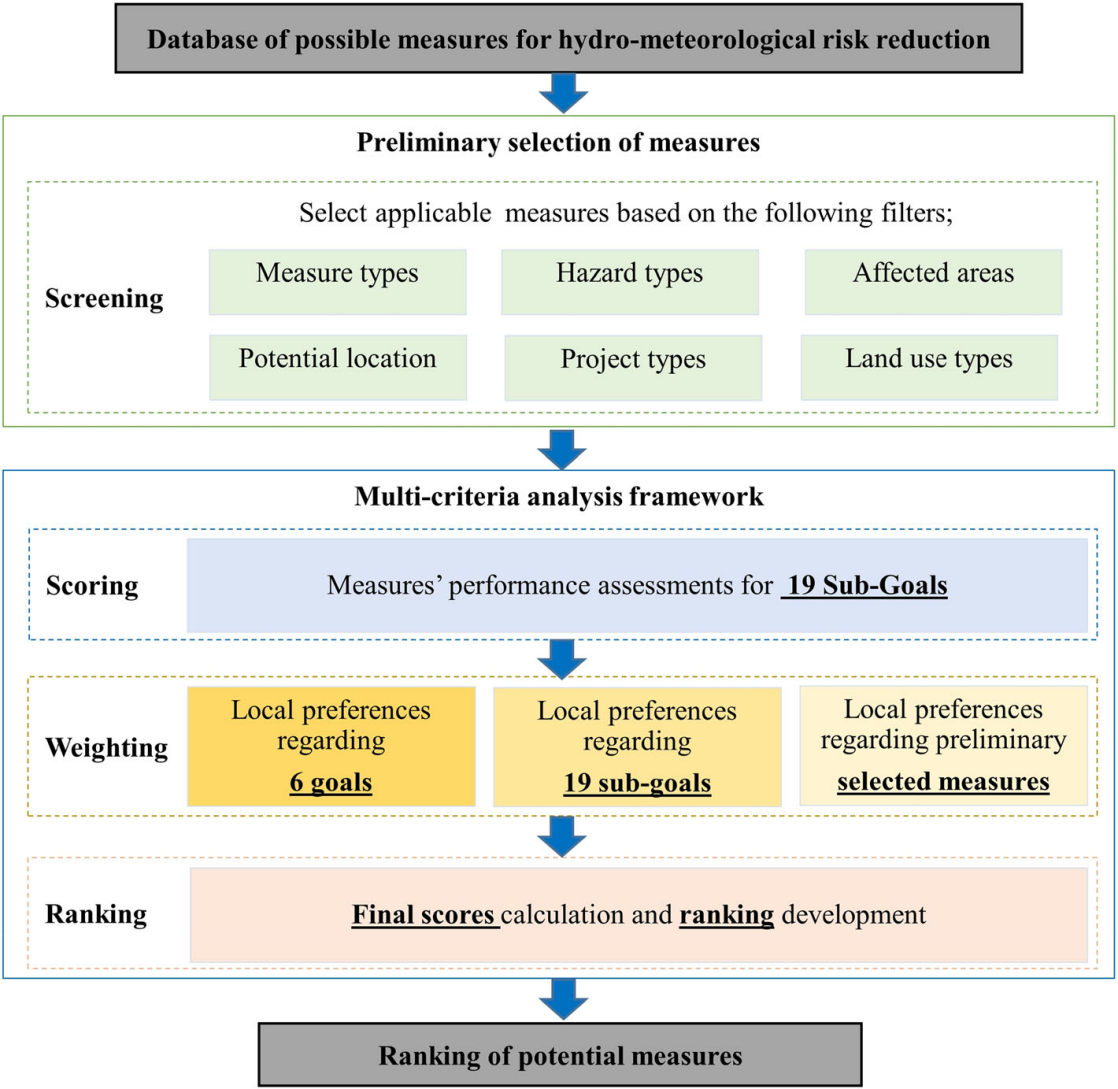
Proposed methodology for selecting potential NBS measures, including preliminary selection and MCA framework

The methodology consists of two steps: the preliminary selection of measures and the multi-criteria analysis framework, as shown as in Fig. 1. This figure presents the different steps of the methodology that the decision-maker needs to follow to select the most suitable measures. This should be applied in the first stage of the planning process to restrict the choice of appropriate measures according to the problems and objectives of a project. The subsequent sections describe the preliminary selection of measures (screening), followed by the criteria chosen for the MCA framework and the processes in this framework (i.e. scoring, weighting and ranking, as shown in Fig. 1).

### Preliminary selection (screening)

The database is developed in this study to provide an extensive list of measures for hydro-meteorological risk reduction. From this list, suitable options for a specific situation need to be singled out. Since not all measures are suitable for all locations and all hazard types, six filters are used in this process to narrow down the list of measures (Fig. 1). The first filter is the measure type, which can be NBS or grey infrastructure.

The second filter is hazard type, as the consequences of an event vary greatly depending on the hazard (e.g. floodplain restoration is suitable for fluvial floods but not pluvial floods). Considered hazard types include pluvial flooding, fluvial flooding, coastal flooding/storm surges, flash flooding, droughts and landslides.

**Fig. 2:**
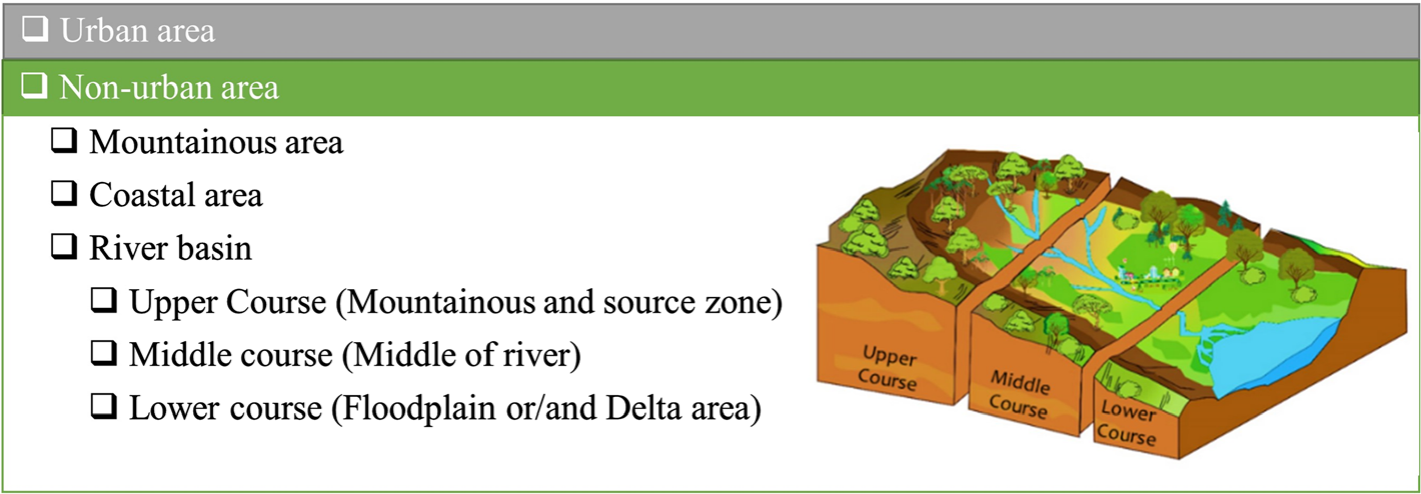
Example of filter (Potential location) with optional sub-filters

Thirdly, the affected area of such problems must be defined as either urban area, non-urban area or both. In the fourth filter, the users identify the potential location for implementation of measures. There are two main types of locations for implementation: urban areas and non-urban areas. Non-urban areas include mountainous area, coastal area and river basin. If the case study is a river basin, the location within the basin also needs to be defined as upper course, middle course or lower course (Fig. 2). It should be noted that at this stage no precise location (micro-location) has to be defined.

The fifth filter is the type of project that would be implemented; i.e. whether the completely new measures are to be implemented or existing measures are to be improved. The final filter is the prevalent land surface type in the area (e.g. artificial surfaces, agricultural areas, forest and semi-natural areas, wetlands or water bodies). Within each filter multiple selections can be made, for example, users can include both urban and non-urban measures in the filter. The data can be collected by using the questionnaire in Supplementary 3.1. The questionnaire should be given to technical stakeholders in the area as it requires technical knowledge.

### Multi-criteria analysis

#### Framework

The innovative stakeholder preference process has been built into the Multi-Criteria Analysis (MCA) framework of the proposed methodology. MCA is a framework for ranking the overall performance of decision options against multiple objectives, which can be used to support complex decision-making situations. MCA is used in this study to select and rank NBS measures as it has the ability to integrate and overcome the differences between technical and social approaches (Loc et al. 2017). MCA also allows for the assessment of possible measures with diverse criteria defined by different units, both quantitative and qualitative.

The most common MCA method that has been used in flood risk management is the Analytical Hierarchy Process (AHP), which is a relatively flexible and easily applicable method (De Brito and Evers 2016). However, in this type of MCA, only a limited number of alternatives can be considered at the same time because AHP uses pairwise comparisons, in which each criterion is compared to the others (Vaidya and Kumar 2006; Guarini et al. 2018). In the proposed framework, there are 25 criteria (see section “Criteria used in MCA framework), thus, AHP is not suitable, since the large number of possible comparisons would increase the process length and complexity for the user.

The MCA in this research is based on the weighted summation method (or linear additive model), which is a special form of Multi-Attribute Value Theory (MAVT) (Belton 1999). For clarification, the following components of the weighted summation method used in this research are defined here:

- Measure: a potential NBS or grey infrastructure measure obtained after the screening process,
- Criteria: potential impacts used to evaluate measures; in this case criteria are being referred to as goals and sub-goals,
- Scores: values used to quantify the performance of each measure in meeting each sub-goal.
- Weights: values given by stakeholders to indicate the importance of each goal, sub-goal and measure,
- Weighted scores of sub-goals/goals: for each measure, this is the sub-goal/goal score multiplied by its weight obtained after processing stakeholder weighting results,
- Criteria score: for each measure, this is summation of all the weighted goal scores,
- Final scores: for each measure, the final score is obtained by multiplying the criteria score by the measure weight.

There are many benefits in using weighted summation. Firstly, it makes the ‘incomparable’ attributes comparable and prioritises them by assigning weights. The ranking can be obtained by multiplying each score (i.e. level of potential impacts) by its weight, followed by summing the weighted scores of all criteria. This process provides not only a ranking of the measures, but also clearly shows strengths and weaknesses of the measures. Secondly, weighted summation provides transparency to the evaluation process due to its simplicity (Marttunen et al. 2015; Guarini et al. 2018). Therefore, the method is very suitable to be used in participatory processes.

In this framework, we can combine stakeholders’ opinions and preferences (weights) with the potential impacts of NBS (scores) in the ranking of measures. The weights are assessed from a survey of relevant stakeholders in participatory processes. The scores have been collected to quantify the performance of each measure based on the literature and expert judgement. Based on this ranking, the decision-maker takes a decision on which measures will need to be further analysed in detail.

#### Criteria used in MCA framework

In order to address the impacts of implementing NBS measures, it is necessary to define criteria taking the primary risk reduction benefit into account, as well as the social, economic and environmental implications of the measures (Boruff et al. 2005). In this framework, the criteria are based on those defined in the RECONECT indicator framework, which itself was derived from existing studies (Raymond et al. 2017). The criteria are referred to as goals and sub-goals in this framework, since they have a hierarchical structure, see Table 1.

**Table 1:** Hierarchical structure of criteria in MCA

Goals	Sub-goals
Hydro-meteorological risk	{Type of Risk reduction that corresponds to selected hazard}*
Water quality	Improve water quality in rivers/watercourses, lakes/ponds
	Improve coastal water quality
	Improve groundwater quality
Habitat structure	Increase habitat area (quantity)
	Habitat provision and distribution (quality)
	To reflect ecological status and physical structure of habitats
Biodiversity	Change in land use
	To maintain and enhance biodiversity
	Reduce disturbance to ecosystems
Social-economic	Increase recreational opportunities
	Education and awareness about NBS
	Maintain and if possible enhance cultural values
	Accessibility
	Improve community cohesion
	Encourage new business models and other community benefits
	Stimulate/increase economic benefits
Human well-being	Direct health and well-being impacts
	Indirect health and well-being impacts

Remark *The HM risk sub-goals depend on the hazard type in the preliminary selection

The goals include hydro-meteorological risk reduction, water quality, habitat structure, biodiversity, socio-economics and human well-being. These 6 goals are further divided into 19 sub-goals. All of these criteria are relevant for future NBS studies, but the specific type of hydro-meteorological risk may change depending on the area. For example, if pluvial, fluvial or flash floods are selected as the hazard type in the preliminary selection, then flood risk reduction will be the sub-goal. The reason that the other sub-goals remain unchanged is that they relate to co-benefits and are, therefore, applicable to all case studies.

Both goals and sub-goals (for which descriptions are given in Tables S1.1 and S1.2) are weighted by stakeholders, but only the sub-goals are used to assess qualitative performance with measures (scoring). These are described in the following sections.

#### Potential impact assessment (scoring)

Potential positive and negative impacts of measures on specific sub-goals are assessed by giving a score to reflect the performance of the sub-goals. The scoring is based on converting qualitative and quantitative data (obtained from a literature review and expert judgement) into a standard scoring system for different sub-goals (see Table 2). The reason for this is that standardised quantitative data are required for the weighted summation method.

**Table 2:**
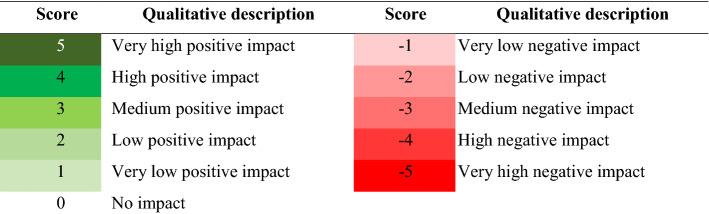
Score level with its qualitative description

The key resources used to assess the qualitative measure performance include reports, online guides, online tools, case studies and scientific articles (Woods Ballard et al. 2007; Klijn et al. 2013; CIRIA 2014; The River Restoration Centre 2014; NWRM 2015; Woods Ballard et al. 2015; WRT 2016; Alves et al. 2018a, b; Bilodeau et al. 2018; Van Coppenolle et al. 2018; Leonardo Mantilla Niño 2019; Watkin et al. 2019; UNaLab 2020). Some resources include very detailed information on potential impacts of specific measures. For example, The EU Natural Water Retention Measures project has published a series of benefit tables for different types of NBS measures (i.e. agricultural, forest, hydro-morphological and urban) in terms of ecosystem services, policy objects and biophysical impacts (NWRM 2015).

In this study, the potential impacts for each sub-goal have been assessed by using indicators (see list in Table S2), then averaging them to their sub-goal. The assessment was generated by assigning a score based on the qualitative descriptions. Scoring of criteria is performed as follows: 5 (Very high positive impact) to 1 (Very low positive impact); 0 (No impact) and − 1 (Very low negative impact) to − 5 (very high negative impact), as shown in Table 2. For example, if there is a very high negative impact in habitat area, this is given a score of − 5, but if the measures can significantly improve or extend the habitat area, this is given a score of 5. These score levels were used to build a performance metric for each measure and each sub-goal.

#### Preferences (weighting)

Since the criteria are not always equally important, a weighting can be attributed to each criterion considered to reflect the degree of its importance. Applying the weighted summation method is only possible if information about the priorities of criteria is available.

Weighting is based on the direct rating method. Usually, the direct rating method uses the judgement of participants/stakeholders, who associate a number in the 0–100 range with the value of each option on the criterion (Dodgson et al. 2009). However, to make this process simpler and easier for participants, they only need to choose weight from 0 to 10 for each criterion and measure. Weight 0 indicates that the criterion is insignificant and can be ignored, weight 5 suggests that it is relatively (moderately) important, and 10 represents the most important criterion among all criteria considered. After the stakeholders give the weights to the criteria, the weights are normalised to have the sum of the weights of each goal equal to one.

In this framework, the weighting is conducted in three steps. Firstly, the stakeholders give their preferences with respect to the six main goals. Then, they give the weights to the 19 sub-goals (section “Criteria used in MCA framework”). Lastly, the stakeholders select which measures are more suitable or applicable to implement. For example, if detention ponds have a high potential for implementing in the area, the stakeholders could give a weight of 9, but if there is no space and this measure is not suitable, the stakeholders could give a weight of 0. The weights for goals and sub-goals can be obtained by using the questionnaire in Supplementary 3.2 on different groups of stakeholders, while the weights on applicable measures can be obtained by using the questionnaire in Supplementary 3.3. There are different methods that can be used to collect the questionnaire responses, such as workshops, digital questionnaires (Microsoft Word) or online survey platforms (Google Forms, Survey Monkey). To obtain the ‘overall’ weight for a criterion from several stakeholders, one can organise group discussions to try to get consensus or to average the weights from the different stakeholders.

#### Prioritisation (ranking)

The last step of the framework is the prioritisation of measures through ranking (see Fig. 1). Ranking of the measures is based on their final score, which is the result of the weighted summation method. After assigning scores for each sub-goal to all the measures (section “Potential impact assessment (scoring)”) and computing the weights for each sub-goal by compiling stakeholders surveys (section “Preferences (weighting)”), the ranking based on the weighted summation method can be calculated by following these steps below.

Firstly, all the assigned weights for both sub-goals and goals need to be normalised on a scale from 0 to 1 (Eq. 1). This is done in order to keep the weights logically distributed.

1 $$W_{i} = \frac{{\omega_{i} }}{{\sum \omega_{i} }}$$

where *W*_*i*_ is normalised weight so that $\sum W_{i} = 1$, and $\omega_{i}$ is the original weight given to the goal and sub-goal (*i*).

Secondly, the score of each measure (*m*_*j*_) for each goal *S*_goal_(*m*_*j*_) can be calculated as the summation of all the weighted sub-goal scores related to that goal (Eq. 2).

2 $$S_{\text{goal}} \left( {m_{j} } \right) = \mathop \sum \limits_{i = 1}^{N} W_{{{\text{subgoal}}_{\text{i}} }} S_{{{\text{subgoal}}_{i,j} }}$$

where *N* is a number of sub-goals within the goal, $W_{{{\text{subgoal}}_{i} }}$ is the normalised weight for sub-goal (*i*) and $S_{{{\text{subgoal}}_{i,j} }}$ is the score for sub-goal (*i*) for measure *m*_*j*_.

Thirdly, the score of each measure (*m*_*j*_) accounting for all criteria (*S*_criteria_ (*m*_*j*_)) can be calculated as the summation of all the weighted goal scores (Eq. 3).

3 $$S_{\text{criteria}} \left( {m_{j} } \right) = \mathop \sum \limits_{k = 1}^{L} W_{{{\text{goal}}_{k} }} S_{{{\text{goal}}_{k,j} }}$$

where *L* is a number of goals, $W_{{{\text{goal}}_{k} }}$ is the normalised weight for goal (*k*) and $S_{{{\text{goal}}_{k,j} }}$ is the score for goal (*k*) for measure *m*_*j*_.

Next, the positive value of *S*_criteria_(*m*_*j*_) is normalised to take values between 0 and 1 (5 is the maximum criteria score); however, negative scores are given a value of 0 (Eq. 4). The reason for this is that only measures that have a positive impact will be considered, while the other measures will be omitted from further analyses for decision-making.

4 $$S_{{{\text{criteria}}_{\text{normalised}} }} \left( {m_{j} } \right) = \left\{ {\begin{array}{*{20}c} {\frac{{S_{\text{criteria}} }}{5}\quad {\text{if}} S_{\text{criteria}} \ge 0 } \\ {0\quad {\text{if}} S_{\text{criteria}} < 0 } \\ \end{array} } \right.$$

The last step is to calculate the final score for each measure, *S*_final_(*m*_*j*_), based on which the measures will be ranked. This can be obtained by multiplying the $S_{{{\text{criteria}}_{\text{normalised}} }} \left( {m_{j} } \right)\varvec{ }$ by measure weights *W*(*m*_*j*_), which have also been normalised (Eq. 5)

5 $${\text{Score}}_{\text{final}} \left( {m_{j} } \right) = S_{{{\text{criteria}}_{\text{normalised}} ,j}} W_{j}$$

This additional step is intended to prevent the selection of a measure that might still not be suitable for the area of interest or might not be accepted for local community.

## Case studies

### General information of the case studies

The methodology can be used for selecting both NBS measures and the combination between NBS and grey infrastructure, for different hazard types and spatial scales. The methodology is here applied to the selection of NBS measures for fluvial flooding at river basin scale in two case studies of RECONECT projects, namely the Tamnava river basin in Serbia and the Nangang river basin in Taiwan.

**Fig. 3:**
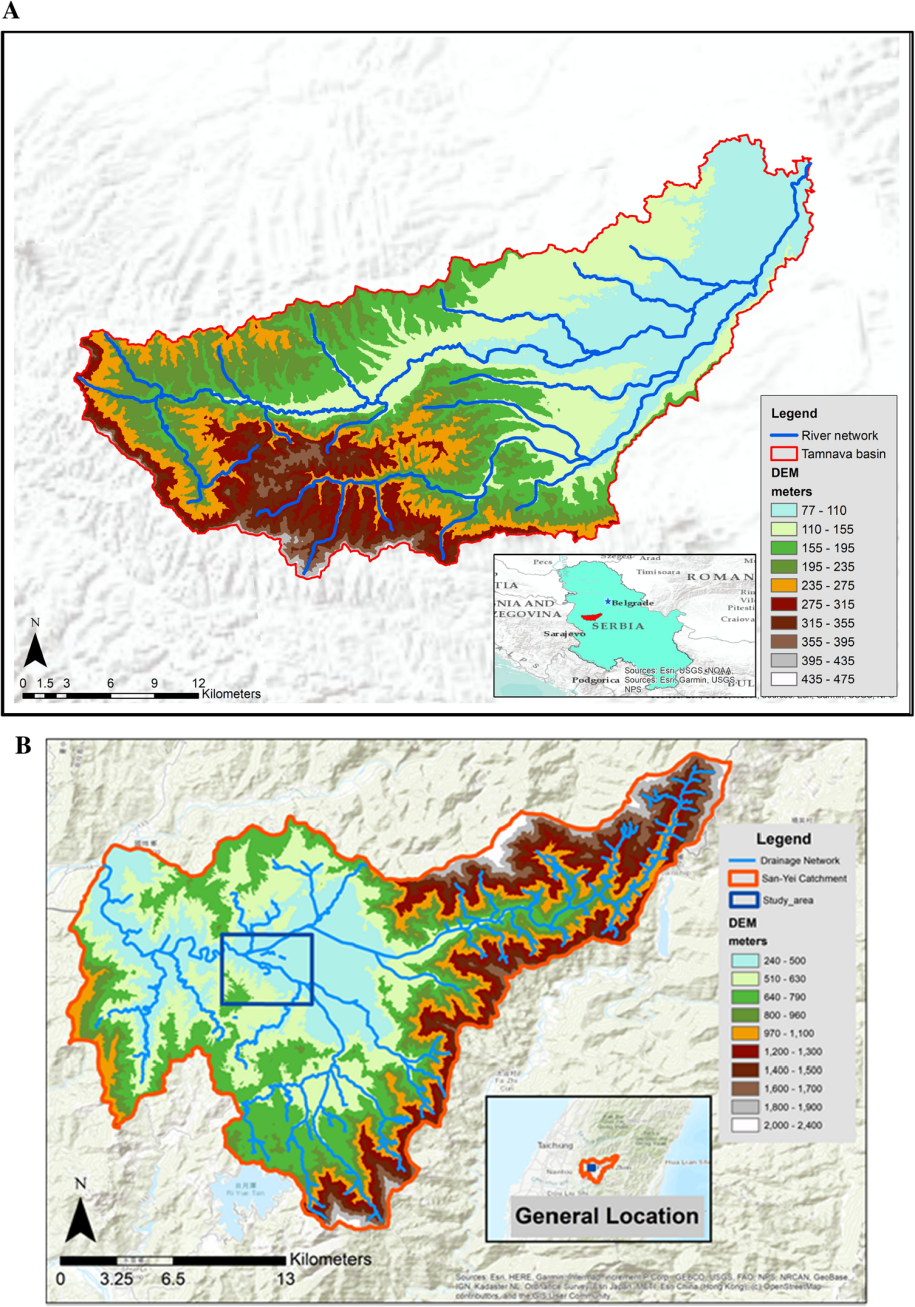
Location of the case studies: Tamnava river basin, Serbia (**a**) and Nangang River basin, Taiwan (**b**)

The Tamnava catchment, located in western Serbia, is a sub-catchment of the Kolubara river and covers an area of 730 km^2^ (Fig. 3a). The Tamnava basin contains two main rivers, the Tamnava and the Ub. The Tamnava river originates in hilly regions (altitudes 400–450 m.a.s.l.), flowing in the middle course through a mildly steep area while the downstream reach of the river is mostly flat. The land use in the catchment is mainly agricultural and residential area. The most significant recent floods occurred in 1999, 2006, 2009 and 2014. In 1999, 6000 ha of land were flooded, and 480 residential buildings and 2050 inhabitants were affected. In 2006 and 2009 similar events with similar consequences occurred. The most severe problems were caused by the flood in May 2014, when the population, economy, infrastructure and natural resources along Tamnava and its tributaries suffered enormous damage (Stanić et al. 2018). Therefore, strategies to reduce flood risk level and the impacts of extreme events are needed.

The Nangang catchment, located in central Taiwan, is a sub-catchment of Dadu River Basin. The Nangang catchment is surrounded by mountainous terrain (altitudes > 1000 m.a.s.l.) with a catchment size of around 440 km^2^. The mainstream part of the catchment is prone to landslides and flooding caused by heavy rainfall. The land use in the catchment is mainly agricultural and residential. Huge damages and loss of lives were recorded during Typhoon Toraji (2001) and Typhoon Kalmaegi (2008). The study area of focus is located at the Niuxiangchu levee system (see Fig. 3b). The studied river reach is roughly 4 km, and the channel is shallow and narrow, which causes high flow velocity and often leads to inundation and riverbank erosion. Since the study area is close to one of the largest cities in the area and frequently suffers from inundation, measures for reducing hazard risks are required.

### Data collection for the case studies

Data collection in this study is based on Microsoft Word and Google Forms questionnaires. The data collection consists of 3 types of questionnaires: (1) questionnaire for collecting local information for preliminary selection of measures (Supplementary 3.1), (2) questionnaire for collecting goals and sub-goals weights (Supplementary 3.2) and (3) questionnaire for collecting weights on applicable measures (Supplementary 3.3). All questionnaires were sent to RECONECT partners in the case studies. Both case studies used the same questionnaires and both partners for the case studies are academic institutions who collaborate closely with stakeholders in their area.

The questionnaire for collecting local information (Supplementary 3.1) was filled in directly by the local RECONECT partners in May 2019 for the Serbia case, and in October 2019 for the Taiwan case. The partners were selected due to their technical knowledge of the case studies.

After that, the local partners explained the purpose of the questionnaire on goal and sub-goal weights (Supplementary 3.2) to respective stakeholder organisations in their case studies (e.g. academia, civil society/NGO’s, local authorities, citizens and political representatives), as well as how it technically should be filled in. The questionnaire was then sent out to those organisations to get a set of responses for that particular case study. In the end, there were two sets of responses from the two case studies addressed in the present work.

After the preliminary selection analysis was performed using the results of the first questionnaire, the questionnaire for collecting weights on applicable measures was developed (Supplementary 3.3). The local partners sent this questionnaire to technical stakeholders (e.g. academia and local authorities) to fill in.

## Results

### Application of the preliminary selection

The database contains, in total, 78 NBS and grey measures, which can be used for the reduction of hydro-meteorological risks. A preliminary selection of potential measures for each case study was performed to define potential measures based on hazard type, affected area, potential location and land surface type, as shown in Table 3. This information was provided by the local RECONECT partners, as explained in section “Data collection for the case studies”.

**Table 3:** Local information that is used as input for preliminary selection

Filters	Tamnava river basin	Nangang river basin
Type of measures	Nature-based Solutions	Nature-based Solutions
Hazard type	Fluvial flooding	Fluvial flooding
The affected area	Urban and non-urban area	Urban and non-urban area
Potential location	Non-urban area: upper course and middle course of river basin	Non-urban area: middle course of river basin
Project type	Implementation of new measures Improvement of existing measures	Improvement or expansion of existing measures
Land surface	Agriculture areas/forest and semi-natural areas/water bodies	Agriculture areas/water bodies

This table shows that the potential location, project types and land surface types are different between the two case studies. Therefore, these different inputs lead to different results of the initial measures selected for the two basins. The selected filters resulted in 18 measures for the Tamnava river basin and 12 for the Nangang river (Table 4). These measures were considered in the MCA.

**Table 4:**
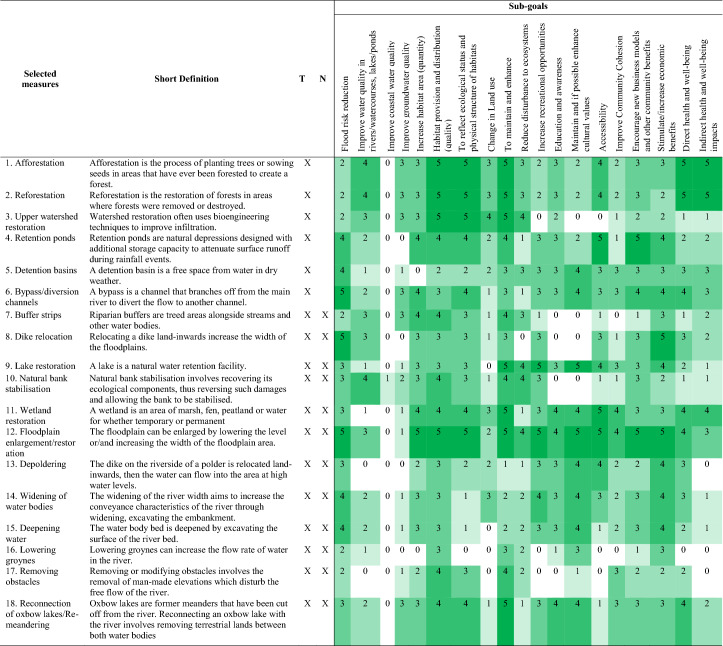
Preliminary selected measures (X) with short definition and scoring of each sub-goals for Tamnava river basin (T) and Nangang river basin (N)

Table 4 also shows the assigned potential impacts (scoring) of filtered measures that have been used for MCA (as explained in section “Potential impact assessment (scoring)”).

### Application of the multi-criteria analysis

#### Criteria weights

The criteria weights for the goals and sub-goals were derived based on stakeholders’ opinions and judgements. These weights identify the importance of the main benefits and co-benefits of NBS measures in the area, and can also represent the trade-offs between NBS benefits. The weights were collected based on the questionnaires in Supplementary 3.2 as explained in section “Data collection for the case studies”. The data collection was done online since it was not possible during this study to organise a face-to-face workshop. There were four responses from academic and local authorities in Serbia, while ten responses were received from academia, civil society/NGO’s, local authorities, citizens and political representatives in Taiwan. The average weight of these responses has been used as the “overall” weight for a criterion from the individual weights of stakeholders in questionnaires.

**Fig. 4:**
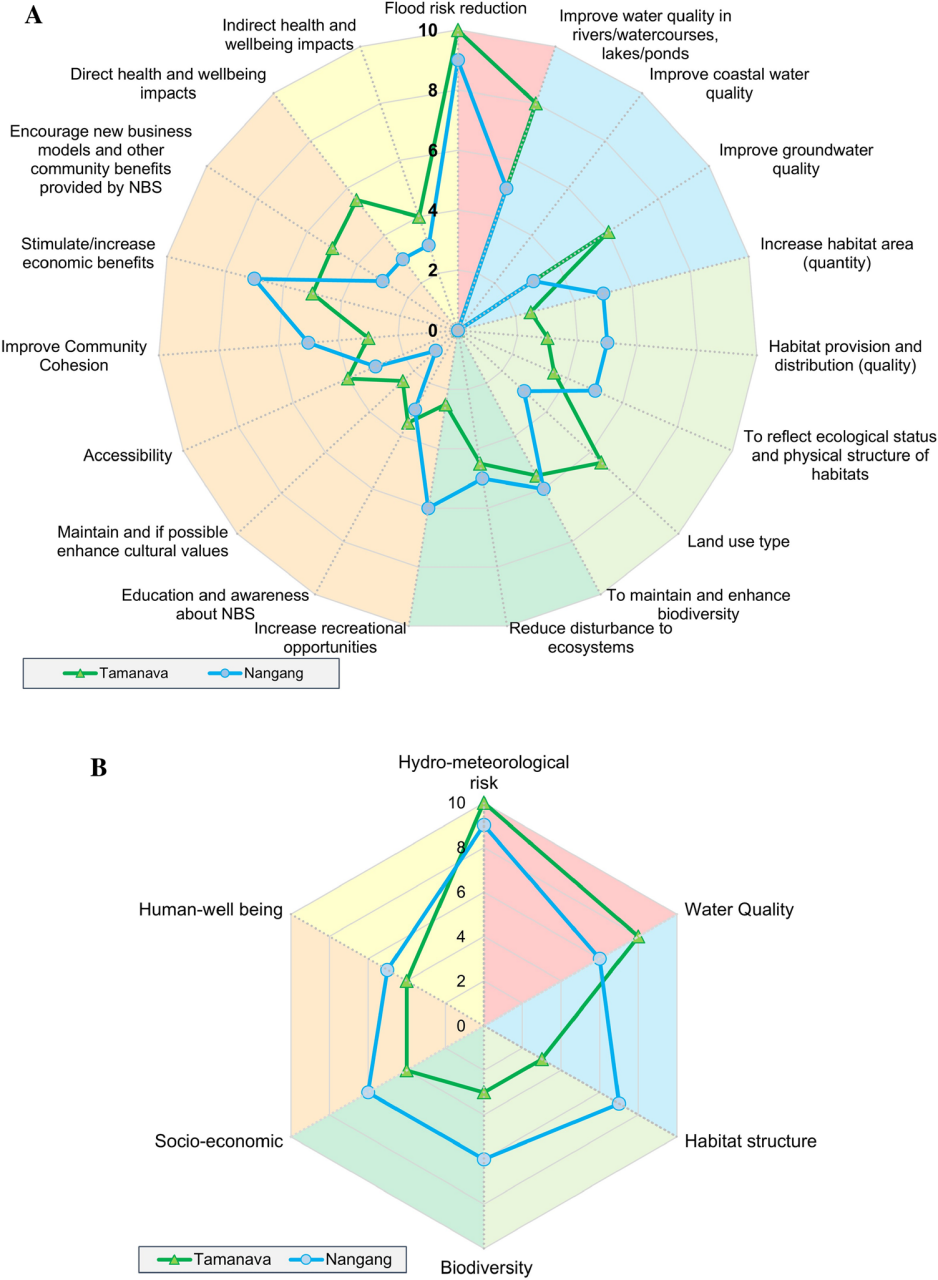
Weighting results of Tamnava and Nangang case studies. **a** Relative importance of evaluating sub-goals and **b** relative importance of evaluating main goals

The assigned overall weight for sub-goals and goals in the two basins is shown in Fig. 4a and b, respectively. The possible range for the weights is from zero (i.e. not important) to ten (i.e. the most important).

In relation to the relative weights among main goals, hydro-meteorological risk reduction is the most important benefit for both case studies (Fig. 4b). A lower weight was given to co-benefits such as enhancing habitat structure, improving socio-economic, whereas a higher importance was given to water quality. The lowest weight was given to human well-being impacts, as it is not their priority for the case studies.

For the weights of sub-goals related to water quality, the most important benefit is to improve surface water quality, while the coastal water quality is not important as both case studies are not close to the coastal area. The weight for water quality is high for Tamnava because there is an intensive use of pesticides in agriculture and coal mining that deteriorate water quality. From the results, it can be seen that the given weight is sensible.

For habitat structure enhancement, changes in land use types are the most important factors for the Tamnava river basin, but the least important for the Nangang river. Among socio-economic benefits, simulate/increase economic benefits was given the highest weight because the stakeholders think that a better economy will help flood risk reduction, since current state of the economy is insufficient to assure satisfactory level of risk reduction.

Comparing the results between the two case studies, the assigned weights have a similar pattern for both sub-goals and goals. However, for the Tamnava case, higher weights are given to the ‘reduce flood risk’ and ‘improve water quality’ goals than Nangang, and lower for the goals related to enhancing habitat structure, biodiversity, socio-economy and human well-being (Fig. 4b). Importantly, for both cases, the weights for goals and corresponding sub-goals are consistent as shown in Fig. 4 (relationship between goals and sub-goals is shown in Table 1). It should be noted, however, that comparing the results from the two case studies is made difficult due to the limited number of responses.

#### Criteria ranking

**Fig. 5:**
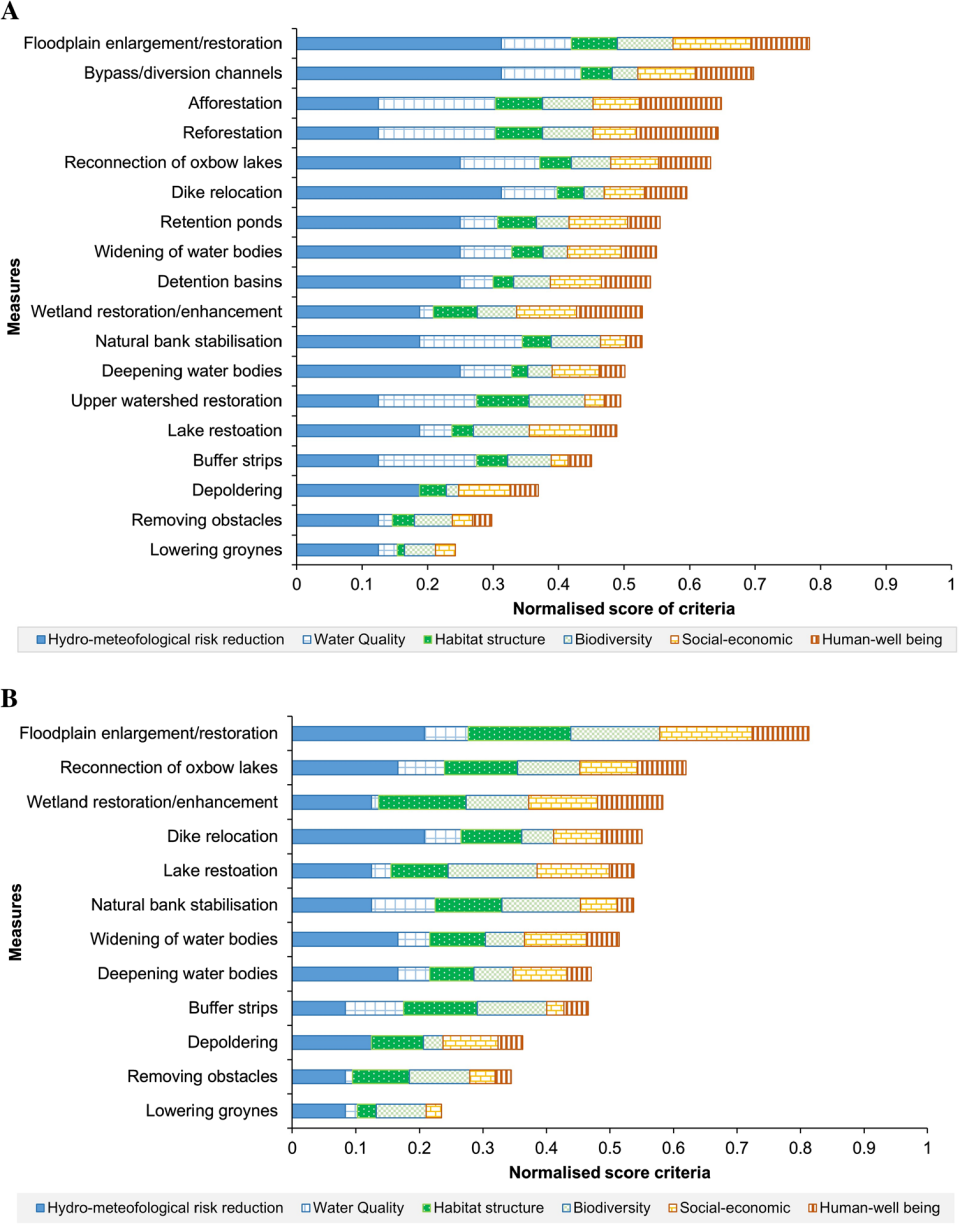
Criteria score and ranking of measures for case studies: Tamnava river basin, Serbia (**a**) and Nangang River, Taiwan (**b**)

The ranking of the measures was performed as the weighted summation of criteria score based on their previously assigned sub-goal scores (as explained in section “Potential impact assessment (scoring)” and Table 4) and the average weights collected from the stakeholders (section “Criteria weights”). The normalised criteria scores and their relative ranking of measures in both the Tamnava and Nangang basins are shown in Fig. 5. This figures also shows the potential benefits, co-benefits and trade-offs of NBSs.

From the ranking of both case studies, it can be observed that floodplain enlargement/restoration has the highest score, as it can provide a number of benefits, including increased flood storage, clean water and open space for recreation, wildlife habitats and biodiversity. On the other hand, measures that work on obstacles (i.e. removing obstacles and lowering groynes) are scored relatively low, as they cannot provide as many co-benefits as other measures.

The measures that are only applicable to Tamnava score highly on co-benefits, especially the measures that can provide a high positive impact on water quality (such as reforestation and afforestation), which is seen as an important benefit for the area (Fig. 5a). Reforestation and afforestation are also able to provide high positive impacts for human well-being, because trees can help to increase mental well-being, reduce chronic stress, mitigate the heat island effect and improve air pollution (Wheeler et al. 2010; Raymond et al. 2017). However, these co-benefits require a trade-off with flood risk reduction.

For the Nangang case study, the measures that can provide multiple benefits score highly (such as reconnection of oxbow lakes, wetland restoration and lake restoration), as the co-benefits are seen as relatively important (Fig. 5b). The benefits provided by wetland and lake restoration to people are: flood risk reduction, water quality improvement, habitat for wildlife, biodiversity support, recreation and aesthetics. Wetland ecosystem services also have a positive interaction to 10 Sustainable Development Goals (SDGs) (Seifollahi-Aghmiuni et al. 2019).

The results also show the advantage of including additional criteria (co-benefits), apart from risk reduction. For example, if only risk reduction is considered, measures like dike relocation will have a higher rank than afforestation for Tamnava and wetland restoration for Nangang, despite having very little co-benefits.

#### Prioritisation of measures

**Fig. 6:**
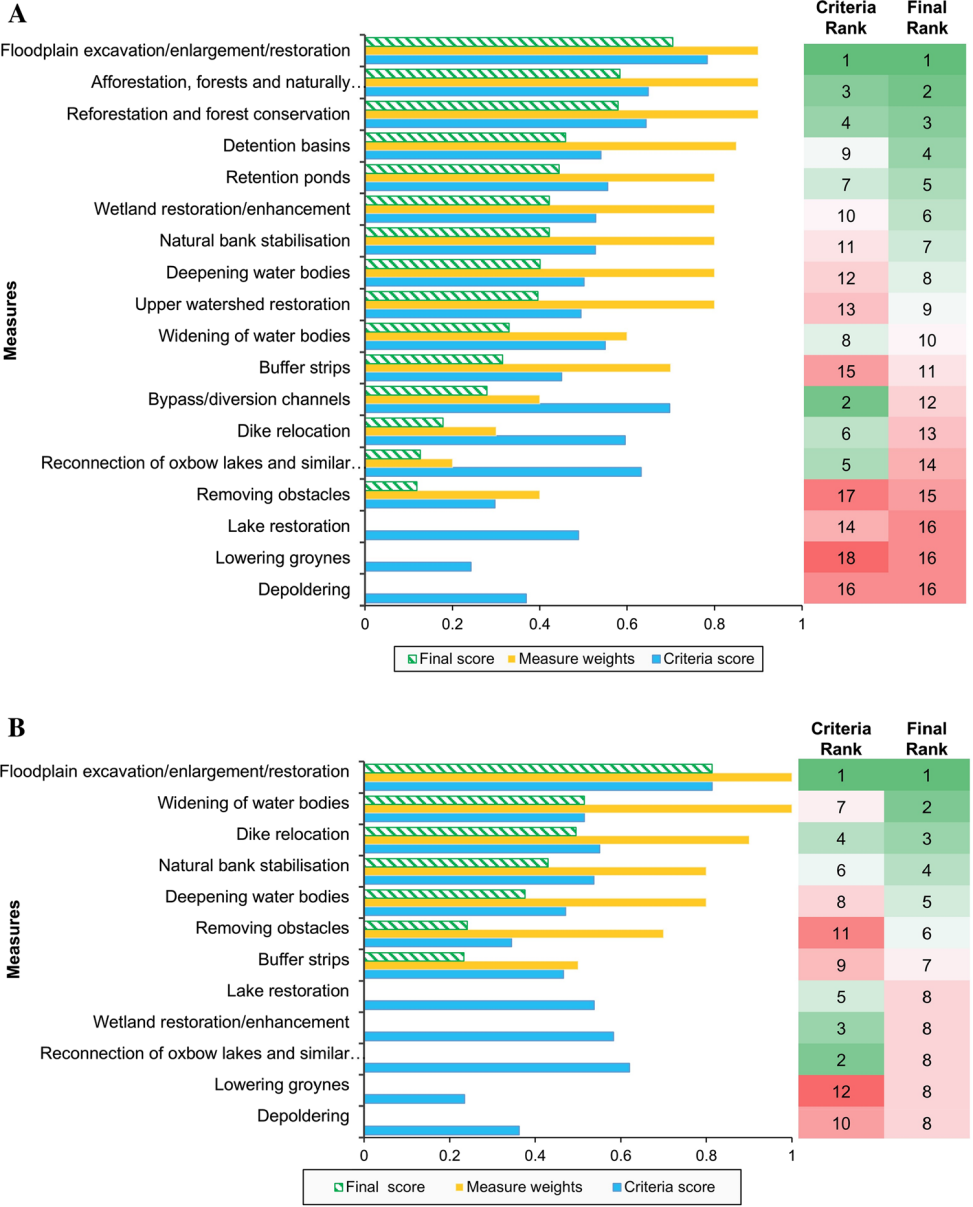
Final ranking of measures based on measures weights of the case studies: Tamnava river basin, Serbia (**a**) and Nangang River, Taiwan (**b**)

This section shows the prioritisation of measures, which is based on their final ranking. The ranking of the measures was performed based on their normalised criteria score (section “Criteria ranking”) and the average measure weights collected from the technical stakeholders. In Serbia, these technical stakeholders were a local authority and an academic institution, and in Taiwan the only response came from academia. The influence of the stakeholders’ weights on the final ranking is also shown. In order to compare the criteria score and final score, both were normalised on a scale of 0–1 and shown in Fig. 6 with their relative ranking.

Floodplain excavation/restoration can be seen as the best solution for both case studies, with and without stakeholders’ preferences. Some measures are not possible to implement in the area, such as depoldering (since there are no polders present in the area) and lowering groynes (there are no groynes present).

Figure 6 a shows the final ranks of the measures for the Tamnava river basin. It can be observed that the measures involving existing features have a lower rank when measure preferences are included. On the other hand, the measures that need completely new implementation, like a bypass channel, now rank high. The reason for this is that in the current situation there are no possibilities for implementing such a measure in the catchment.

For the Nangang river, it can be seen that 5 measures out of 12 are not suitable (see Fig. 6b), even though they perform well based on the criteria ranking. This is due to the fact that the case study area does not currently have the associated features (groynes, lakes etc.). This result, therefore, shows the importance of including the preference measures into the analysis.

## Discussion

From the above results, it can be seen that the preliminary selection process can help to eliminate the measures that are not relevant to the problem, location or characteristics of the area. This process is important in identifying potential NBSs that are suitable to the project (Romnée and De Herde 2015; Zhang and Chui 2018).

The multi-criteria analysis framework used to select NBS measures for river basin scale considers a number of criteria categorised under hydro-meteorological risk, water quality, habitat structure, biodiversity, socio-economic and human well-being aspects.

Outcomes of the MCA were derived from previously assigned sub-goal scores and the weights collected from the stakeholders. The results show that the proposed methodology can be used to analyse the performance of measures with a comprehensive and holistic approach, by taking into account not only the primary goal of risk reduction but also related co-benefits such as water quality, ecosystem services, socio-economic aspects, human well-being and economic factors. Moreover, including all these benefits in the framework can help the stakeholders and decision-makers to recognise trade-offs of NBS. In considering trade-offs, risk reduction and ecological and social outcomes need to be acknowledged so that both communities and ecosystems benefit from NBS measures (Brink et al. 2016). Applying the methodology to two case studies proved that MCA is a very good starting point for identifying and ranking measures for reducing risk reduction and enhancing other benefits. Similar results have been obtained by Van Ierland et al. (2013) and Jayasooriya et al. (2019).

From the criteria weights, it can be observed that risk reduction is considered the most important benefit, followed by water quality, whereas biodiversity and habitat structure benefits were not so important for the area. However, this could be a possible bias or uncertainty due to the nature of weighting. The source of this uncertainty could be from their profession. For example, risk managers may give a higher weight for risk than environmental and social benefits, while environmental authorities may think that environmental benefits are more important than risk and social benefits.

Therefore, we recommend decision-making and policy management studies based on a larger sample of stakeholder responses are needed to examine uncertainties in the weights and sensitivity of the final ranking of the measures to the weights assigned by the stakeholders. It would be particularly important to compare responses of different groups of stakeholders, such as local authorities, civil protection or academia. Moreover, analysing the larger sample of stakeholder responses can indicate the needs of different groups, and, hence, facilitate further improvements of the goal/sub-goal list.

The criteria ranking results show the ranking of measures and the potential benefits, co-benefits and trade-offs of NBS. Floodplain enlargement/restoration has the highest scores in the case studies as it can provide multiple benefits, such as giving more room for the river, improving water quality, providing more space for recreation activities, protecting people and properties and enhancing habitat and biodiversity. The results also show that if only risk reduction is considered, measures such as dike relocation or widening of water bodies will have a higher rank, since they have a high positive impact in risk reduction. As a result, it is important to include both main benefit and co-benefits into an analysis so that communities and ecosystems can benefit from selected NBS measures. Similar results have been obtained by Alves et al. (2018a), Kuller et al. (2019). Even though a measure achieves a higher rank for total benefit, attention needs to be paid to the trade-off on risk reduction as it is the main objective for implementing NBS.

In many studies, MCA uses criteria scores for the final ranking or results (Young et al. 2010; Aceves and Fuamba 2016; Loc et al. 2017). However, in this study, stakeholders’ weights on the measures are included into MCA. The difference in the ranking of the criteria scores and final scores provides interesting results. For example, removing obstacles from the riverbed, which is technically viable solution, was disregarded in the final ranking in the Tamnava basin, since effectiveness and benefits from this particular solution were not recognised by the stakeholders. Similarly, lake restoration performed very well in criteria scores, but not in the final score as the measure is not suitable for the case studies. This shows the importance of including this step in the MCA framework. The results also showed that the pre-selection process does not account for local characteristics in detail. Therefore, it might be beneficial for management and decision-making to define the applicability of measures directly after the preliminary selection intended to eliminate non-applicable measures in the analysis.

However, there is a limitation in this final ranking process, which is that giving weights to measures seems more suitable to technical stakeholders than general stakeholders. The reason for this is that giving weights for the measures requires some technical knowledge. Therefore, it may be better to obtain weights from a face-to-face workshop rather than individual questionnaires.

It is also recommended that this methodology should be incorporated into a web-based decision-making tool, providing, therefore, a simple and easy application for users. This has been suggested in a recent review article by De Brito and Evers (2016). Moreover, a spatial allocation method should be developed to define potential specific location for the selected measures. Finally, methods for further evaluation highly ranked measures and combinations thereof should be developed through 1D–2D hydrodynamic models, cost–benefit analysis and optimisation.

## Conclusion

This paper proposes an innovative methodology for selecting potential measures which reduce hydro-meteorological risk as a main objective and simultaneously offer co-benefits. This methodology consists of a preliminary selection of feasible measures for hydro-meteorological risk reduction, followed by a multi-criteria analysis framework. This provides an easy-to-use decision support tool, aimed at planners and decision-makers, which systematically and transparently defines suitable measures.

The methodology presented here upscales from previously developed methods discussed in the introduction. The first improvement consists in the inclusion of different types of hazards and scales (i.e. river basin, coastal zone or urban area) into the analysis. Secondly, this method includes a wide range of possible NBS benefits (reduction of hydro-meteorological risks, improvement of water quantity, protection and enhancement of habitats, safeguard of biodiversity and socio-economic and human well-being). By including these criteria into MCA (Multi-Criteria Analysis), the methodology results in a different ranking of the measures compared to the traditional ranking based on risk reduction alone. Thirdly, it provides the opportunity for decision-makers to define preferences among these benefits. Involving stakeholders in the process of measure selection in an MCA can introduce additional relevant aspects that might be unnoticed by engineers. Lastly, the methodology enables decision-makers to identify the most suitable and preferable NBS measures for the area, which can help obtain more realistic results in relation to suitability of measures to the case studies.

Based on the preliminary selection process, all measures chosen may not be applicable in the study area, as this selection process does not include detailed local conditions. These are taken into account in the MCA phase of the present work, hence the methodology can be used to ensure that the selected measures are quite suitable for the basin of interest.

The application of the methodology to two case studies proved its usefulness for decision-making for river basin planning. It helps planners and decision makes to select potential measures and formulate desirable benefits and co-benefits at the basin scale.

## Electronic supplementary material

Below is the link to the electronic supplementary material.

Electronic supplementary material 1 (PDF 1778 kb)
