# Supplementary material for: Incorporating stakeholders’ preferences into a multi-criteria framework for planning large-scale Nature-Based Solutions
Source: Ambio. 2020 Dec 1;50(8):1514–31. doi: 10.1007/s13280-020-01419-4 (PMC8249655; doi:10.1007/s13280-020-01419-4)
Supplement: Supplementary file 1 — Electronic supplementary material 1 (PDF 1778 kb) [file 13280_2020_1419_MOESM1_ESM.pdf]

Ambio

Electronic Supplementary Material
This supplementary material has not been peer reviewed.

Title: **Incorporating stakeholders' preferences into a multi-criteria framework for planning large-scale Nature-Based Solutions**

Laddaporn Ruangpan, Zoran Vojinovic, Jasna Plavšić, Dong-Jiing Doong, Tobias Bahlmann, Alida Alves, Leng-Hsuan Tseng, Anja Randelović, Andrijana Todorović, Zvonimir Kocic, Vladimir Beljinac, Meng-Hsuan Wu, Wei-Cheng Lo, Blanca Perez-Lapeña, and Mário J. Franca

Table S1.1 List of goals and their description

Goals	Description
Hydro-meteorological Risk	This goal represents the hydro-meteorological risk that is present in the chosen area and reflects the need for reducing it.
Water Quality	This goal indicates the importance of improving the overall water quality, including surface and groundwater bodies in the investigated area. Water quality is an essential factor since it interrelates with other factors such as ecosystems and human health.
Habitat structure	The habitat structure category determines the main aspects related to habitat quantity and quality in the area. This includes the importance of increasing habitat area, habitat provision and distribution, ecological status and physical structure of habitats and land use type.
Biodiversity	The biodiversity refers to the variety of life on earth at all its levels, from genes to the ecosystem. It presents the status of ecosystems and enhances local biodiversity in order to create conditions where various species can thrive in abundance and live without disturbance.
Social-Economic	This goal relates to the people living in the chosen area or those affected by current developments. This category reflects on the need or the present opportunities that are needed to increase socio-economic development in the area. It includes factors such as recreational space, educational opportunities, and community cohesion.
Human well-being	This goal relates to the enhancement of human well-being benefit in the area from implementing NBS. The importance of this category reflects on health and well-being such as mental well-being, physical activities/health, urban heat island effect mitigation, air pollution improvement through capture/removal by vegetation etc.

Table S1.2 List of sub-goals and their description

Sub-Goals	Description
Flood risk reduction in urban areas and around rivers, lakes, watercourses, etc.	This sub-goal considers the importance of flood risk reduction in the area. This includes urban areas, rivers, lakes and watercourses. It also means to reduce flood vulnerabilities, reduce flood hazards, reduce flood peaks, slow down runoffs and delay flood peaks.
Coastal flood risk reduction	This sub-goal indicates a reduction of the flood risk in the coastal area. Coastal flooding means that a sudden and abrupt inundation of a coastal area occurs. Often this is caused by a short term increase of sea level due to storm surges or extreme tides.
Drought risk reduction	Drought risk reduction refers to various factors such as frequency, duration and time scales of precipitation as well as soil moisture content, storage capacity, available water supply and the water consumption of the area.
Landslide risk reduction	Landslides occur as a process of downwards and outward moving and slope forming materials, such as rock, debris, soil or all combined. They can be triggered by intensive rainfall, earthquakes, or rapid stream erosion. Factors like unstable hillslopes and pressure from urbanisation and deforestation can increase chances of landslides.
Improve water quality in rivers/watercourses, lakes/ponds	This sub-goal indicates whether the improvement of water quality in surface water bodies like rivers and lakes is required. Change in water pollution (i.e., both point sauces and non-point sources), heavy metals, nutrient contamination and sediment deposition, are important key indicators, which can lead towards problems for ecological and human health.
Improve coastal water quality	This sub-goal reflects on the water quality in coastal areas. The main key factor here is the pollution level of coastal waters. Most ocean pollution is influenced by the land, either along the coastline or coming from the. It causes dangerous conditions for marine life and human health.
Improve groundwater quality	Groundwater is a very important source in terms of drinking water supply in many nations. Groundwater quality is less prone to contamination than surface water but can also be affected by various pollution sources and infiltration through the soil. The indicators can be attenuation of pollution in groundwater, change in soil quality or seawater intrusion.
Increase habitat area (quantity)	This sub-goal focuses on the importance of increasing the habitat area (i.e., change in riparian, aquatic, wetland and terrestrial habitats for local species). The size of the habitat has significant effects on reproduction and population persistence for various species. Increasing green space can improve population growth and reduce the risk of species extinction.
Habitat provision and distribution (quality)	Habitat provision and distribution refers to the quality state of a habitat. This can be seen in the distribution of green space in the area and whether habitat structures are showing good connectivity with each other. Better connectivity of habitat structures leads to a higher ecosystem function where higher diversity of animal and plant species can be found, leading to better overall ecosystem health.
To reflect the ecological status and physical structure of habitats	This sub-goal indicates the significance of carrying out conservation and protection strategies to reflect on the ecological status and physical habitat structure of the area. This can be done by monitoring changes of

	vegetation, erosion protection and the overall conservation status and by looking at possible trends and future projections.
Land use type	This sub-goal relates to the type of land that could potentially be used or changed to implement a possible NBS. A low impact space (e.g. rooftop) can be used to increase its values of ecosystem services and functions. Often compromises are needed to prevent conflicts in those areas and provide sustainable solutions.
To maintain and enhance biodiversity	This sub-goal relates to the importance of maintaining and enhancing biodiversity, which means improving number and types of protected species, native species and their density and diversity in general.
Reduce disturbance to ecosystems	Reducing the disturbance to the ecosystem, in this case, means to decrease stressing ecological factors in the area such as specific amounts of non-native/migrated and invasive species that could be a threat to the local ecosystem or local species.
Increase recreational opportunities	Increased recreation opportunities mean providing recreational space, giving the possibility to people to spend their free time in the area, engaging livelihood activities and increasing number of tourists. These can be beneficial for human well-being from both biological and psychological aspects.
Education and awareness	A site can be seen as an opportunity to provide knowledge and awareness about connected key aspects such as the reduction of major risks (e.g. flooding) in the area as well as supporting ecosystems.
Maintain and if possible enhance cultural values	Maintaining and (if possible) enhancing cultural values present in the area includes reducing the risk of losing cultural heritage during extreme weather events. Also, it means to improve working environments for local food production and to maintain or increase numbers of cultural events.
Accessibility	Accessibility reflects on the factor of how easy it is to reach/ access the NBS site. It includes free space of the area and connection between area and surrounded homes.
Improve Community Cohesion	Improve community cohesion is enhanced when the people are engaged in an activity which connects them. This also includes the number of people who communicate with the neighbourhood in the NBS area and cognitive and social development in children and young people.
Stimulate/increase economic benefits	This sub-goal shows the significance of increasing or stimulating economic growth in the area. This includes factors such as avoided future damage costs and reduced costs from energy and carbon saving through NBS (e.g. reduced energy consumption of buildings). Also, factors can be seen in reducing the costs of negative health impacts and an increase in property values.
Encourage new business models and other community benefits provided by NBS	This sub-goal refers to the opportunity of encouraging new business models and community benefits to arrive through a NBS. Due to increasing attractiveness of the area, new businesses could be interested in settling, also through the improvement of living and working conditions. Furthermore, this could mean an increase in created green jobs in the area.
Direct health and well-being impacts	Direct health benefits can be considered in terms of mental and physical well-being. One of the main reasons why an NBS could improve human health can be seen in the fact that it allows increasing physical activity to

	the people who live or visit the area, spending more time outside and being more engaged within the community.
Indirect health and well-being impacts	Indirect health impact can be seen due to improving air quality (e.g. removing pollution with planted vegetation), less noise pollution, possible reduction of heavy metal contents in the environment and the mitigation to the urban heat island effect. NBS can reduce the exposure level of toxic substances towards humans and can be beneficial in terms of human health.

Table S2 List of sub-goals and their indicators that used to assess the performance

Sub-Goals	Indicators that used to assess the performance of sub-goals
**Flood risk reduction**	Surface run-off reduction
	Slowing and storing runoff
	Flood hazard
	Vulnerability
	Delay time to peak
	Flood peak reduction
**Improve water quality in rivers/watercourses, lakes/ponds**	Change in water pollution caused by wastewater (point sources)
	Reduced pollutants coming from land to water (non-point sources)
	Attenuation of heavy metals and nutrients contamination in surface water
	Sediment deposition
**Improve coastal water quality**	Reduction of pollution in coastal waters
	Coastal water pollutants in shellfish
**Improve groundwater quality**	Attenuation of pollution in groundwater
	Change in soil quality
	Seawater intrusion
**Increase habitat area (quantity)**	Changes in riparian habitat
	Changes in aquatic habitat
	Change in wetland habitat
	Changes in terrestrial habitat
	Increase green area
**Habitat provision and distribution (quality)**	Distribution of public green space
	Connectivity/fragmentation of habitat structural
	Change in location of habitat boundaries
**To reflects ecological status and physical structure of habitats**	Change in vegetation along watercourses
	Conservation status of habitats
	Shoreline characteristics and erosion protection
**Land use type**	Low impact space
	Diversity of land use in the agricultural area
	Change in land cover
	Change in land use
**To maintain and enhance biodiversity**	Restricted-range species
	Species richness and composition in respect to indigenous vegetation and local/national biodiversity targets
	Number and type of protected species
	Density of Species
	Diversity of species
	Type, density of native species
**Reduce disturbance to ecosystems**	Number, area, location, of non-native/mitigated animal and planted species
	Number, area, location, of invasive non-native animal and planted species that are threatening to ecosystem, habitats or species
**Increase recreational opportunities**	Increasing recreational opportunities of NBS area
	Number and value of people visit or spend free time in NBS area
	Number of people engaging in alternative livelihood activities in the NBS area
	Number of tourists
**Education and awareness about NBS**	Provision of NBS sites for education and research
**Maintain and if possible enhance cultural values**	Loss of cultural heritage due to hydro-metrological events/ due to land take
	Food production
	Number of cultural events in NBS area

Sub-Goals	Indicators that used to assess the performance of sub-goals
**Accessibility**	Accessible NBS area per capita
	Footpath network recover through erosion reduction and improvement of path smoothness
	Average journey time for people by foot to NBS area or average distance from home/public transportation to NBS area
**Improve Community Cohesion**	The number of people communicate with neighbourhood in the NBS area
	Cognitive and social development in children and young people
	Community development and cohesion
**Encourage new business models and other community benefits provided by NBS**	Number of subsidies or tax reductions applied for (private) NBS
	Number of new businesses attracted from NBS
	Number of green jobs created
	Enhancing attractiveness of places for living and working, and to visit
	Gross value added per employees based on full-time equivalent jobs in the green sector.
	Finances and willingness to pay
	Increased competitive advantage for cities applying NBS
**Stimulate/increase economic benefits**	Reduced/avoided damage cost from hydro-meteorological risk reduction
	Economic benefit from the reduction of stormwater that typicaly needs to be treated in a public sewerage system
	Energy and carbon savings from reduced building energy consumption (heating and cooling)
	Reduce cost of health impacts of air and noise pollution
	Value of reduced CO2 emission and carbon sequestration
	Reduced need for management and maintenance
	Change in land and/or property values
**Direct health and wellbeing impacts**	Mental well-being
	Physical health/activities
	General self-rated health
	Reduction in chronic stress and stress-related diseases
	Reduction in number of cardiovascular morbidity and mortality events Episodes of water-borne diseases
**Indirect health and wellbeing impacts**	Urban heat island effect mitigation
	Increase in pathogen vector habitat
	Change in heavy metal emission
	Avoided greenhouse gas emissions [should this be in Nature section? very long term indirect relationship to human health and wellbeing]
	Air pollution improvement through capture/removal by vegetation
	Noise pollution attenuation

Supplementary 3.1 Questionnaire for collecting local information to use as an input for preliminary measure selection

H2020 project "Regenarating ECOsystems with Nature-based solutions for hydro-meteorological risk rEduCTion (RECONECT)"

RECONECT

INTRODUCTION

Introduction to the project

The **RECONECT project** aims to develop a framework that can help affected people and involved stakeholders to find suitable and sustainable solutions for their area. The project focusses in selectin and evaluating measures that are known as **'Nature-based solutions' (NBS)**. NBS have been proven in many cases that they are beneficial in terms of flood risk reduction and other types of hazards. In comparison to the traditional grey infrastructure, NBS can give the opportunity not only to fulfil one single purpose (e.g. reducing the flood risk) but also to provide valuable co-benefits to the ecosystem and human well-being as well as climate resilience. More information about the project can be found on the website (**www.reconect.eu**).

Survey description

Since not all measures are suitable for all locations and all hazard types, six filters are used in this process to narrow down the list of measures. Therefore, this survey is conducted in order to collect valuable data for these six filters, which will contribute to **Preliminary selection measure** process (See Figure below). The outcome will be seen as suitable measures for a specific situation.

6 filters that are used to narrow down the list of measures that have potential to reduce the local hazards in the particular affected area are described below. The criteria area; **measure type, hazard type, affected area, potential location for implementation of measures, type of project, and land surface type.**

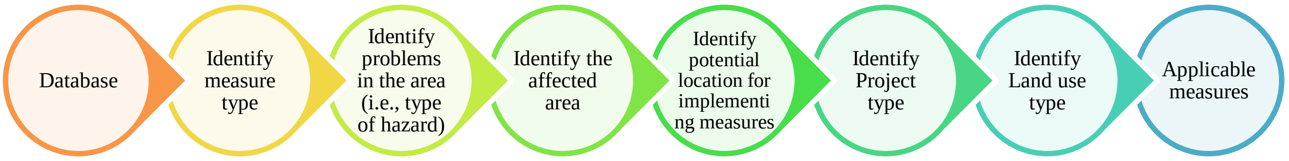

1. Measure type

Please select **type of measures** that you wish to implement in order to reduce local risk (you can select more than one)

☐ Nature-Based Solutions ☐ Grey infrastructures

2. Hazard type

Select hazards that your area have been experienced (you can select more than one)

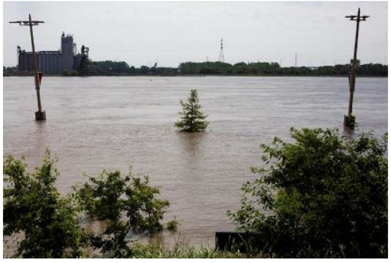

☐ Pluvial flooding

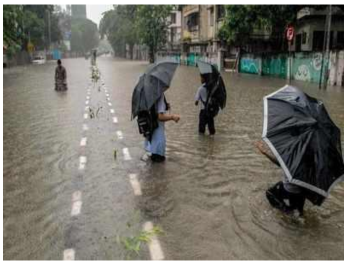

☐ Fluvial flooding

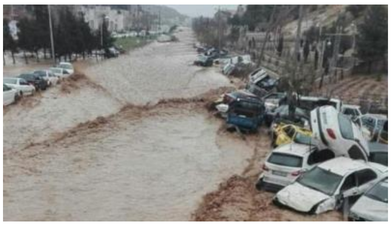

☐ Flash Flooding

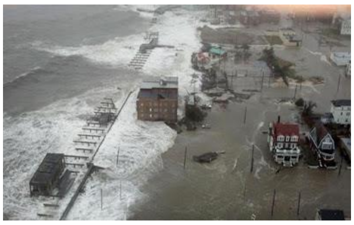

☐ Coastal flooding/storm surge

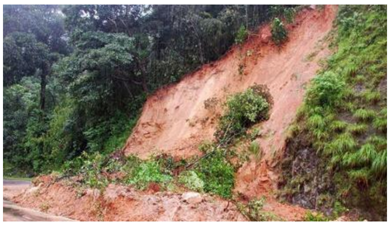

☐ Landslides

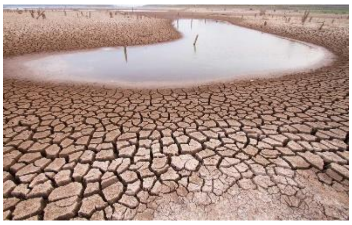

☐ Drought

3. Affected area

Please select **the area** that have been **affected** by the hazard (you can select more than one)

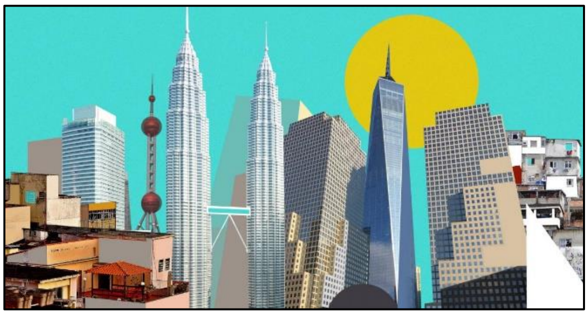

☐ Urban area

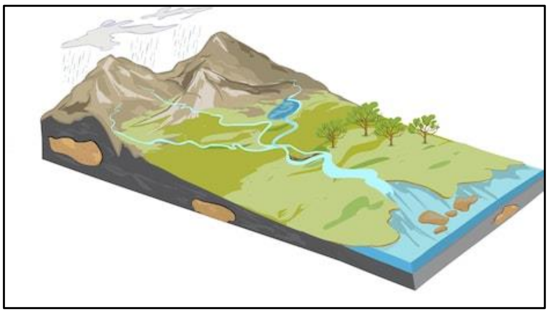

☐ Non-urban area

4. Potential location for implementing measures

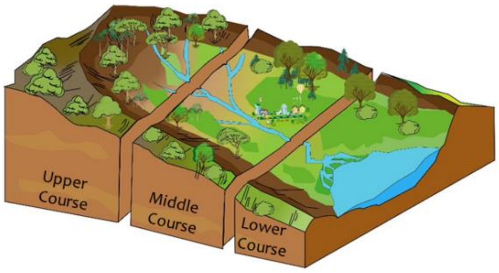

If your area is **urban area** please select ☐

If your area is **non-urban area** please select below (you can select more than one);

- Moutainous area ☐
- Coastal area ☐
- River basin
  - Upper Course (Mountainous) ☐
  - Middle course (Middle of river) ☐
  - Lower course (floodplain and Delta area) ☐

5. type of project

Please select **type of project** that you wish to implement in order to reduce local risk (you can select more than one)

☐ Implementation of new measures ☐ Improvement or expansion of existing measures

6. Land surface type

Please select **Land surface type** that you wish to implement in order to reduce local risk (you can select more than one)

☐ Artificial surface

☐ Agriculture area

☐ Forest and semi-nature areas

☐ Wetlands

☐ Water bodies

Supplementary 3.2 Questionnaire for collecting stakeholders' preferences on goals and sub-goals

H2020 project "Regenarating ECOsystems with Nature-based solutions for hydro-meteorological risk rEduCTion (RECONECT)"

INTRODUCTION

**Introduction to the project**
The **RECONECT project** aims to develop a framework that can help affected people and involved stakeholders to find suitable and sustainable solutions for their area. The project focusses in selectin and evaluating measures that are known as **'Nature-based solutions' (NBS)**. NBS have been proven in many cases that they are beneficial in terms of flood risk reduction and other types of hazards. In comparison to the traditional grey infrastructure, NBS can give the opportunity not only to fulfil one single purpose (e.g. reducing the flood risk) but also to provide valuable co-benefits to the ecosystem and human well-being as well as climate resilience. More information about the project can be found on the website (**www.reconect.eu**).
**Survey description**
This survey is conducted in order to collect valuable data, which will contribute to the of the **feasibility selection process of NBS measure**. Collected inputs will be used for a **Multi-criteria Analysis (MCA)**. The outcome will be seen as feasible measures to discuss possible solution opportunities for the existing problems in the area. This process is done with **multiple stakeholders** in order to reflect on all involved stakeholder opinions and to form a conclusion based on expert knowledge, local opinions, and knowledge from involved people in the area.
**Filling in Weights for Goals/Sub-goals**
In the next section (**Weighting of Goals & Sub-goals**), you will find the Goals & Sub-goals that are included in the NBS selection and evaluation process. You are asked to give weight on those Goals & Sub-goals based on your knowledge of the area and **your opinion of the importance to address the specific Goal or Sub-goal**. Description of Goals & Sub-goals can also be found in the next section.
**Select the group of stakeholder that best describes your position**

☐ Authorities	Name of organization ....
☐ Political Representatives	Name of organization ....
☐ Civil Society Commercial	Name of organization ....
☐ Sector Academia / Research	Name of organization ....
☐ Media	Name of organization ....
☐ International and	Name of organization ....
☐ Utilities	Name of organization ....

Explanation
The six main goals that are part of the NBS evaluation process are described below: **Hydro-meteorological Risk, Water quality, Habitat structure, Biodiversity, Socio-economic and human well-being**. Furthermore, these goals are divided into 19 sub-goals, which are also described below. The provided information of the goal/sub-goal description can be used as a basis to give a reasonable weight for the chosen category.<br><br>Please fill in weight for each category to show the importance of addressing the related issue in the area. Weights should be given by choosing a value from **0 to 10**, where a value of **0** represents **not important,** and a value of **10** means the **most importance**. Also, please give a short explanation of why do you give a certain weight (e.g., drought risk has very low value because this risk is barely present in the area).

Main goals

1. Hydro-meteorological Risk

This goal represents the hydro-meteorological risk that is present in the chosen area and reflects the need for reducing it.

What importance do you place on reducing the hydro-meteorological risk in the area?

Put Weight here (0-10):
Give an explanation of the given weight:

2. Water Quality

This goal indicates the importance of improving the overall water quality, including surface and groundwater bodies in the investigated area. Water quality is an essential factor since it interrelates with other factors such as ecosystems and human health.

What importance do you place on improving the water quality in the area?

Put Weight here (0-10):
Give an explanation of the given weight:

3. Habitat structure

The habitat structure category determines the main aspects related to habitat quantity and quality in the area. This includes the importance of increasing habitat area, habitat provision and distribution, ecological status and physical structure of habitats and land use type.

What importance do you place on improving habitat conditions in the area?

Put Weight here (0-10):
Give an explanation of the given weight:

4. Biodiversity

The biodiversity refers to the variety of life on earth at all its levels, from genes to the ecosystem. It presents the status of ecosystems and enhances local biodiversity in order to create conditions where various species can thrive in abundance and live without disturbance.

What importance do you place on improving biodiversity in the area?

Put Weight here (0-10):
Give an explanation of the given weight:

5. Social-Economic

This category relates to the people living in the chosen area or those affected by current developments. This category reflects on the need or the present opportunities that are needed to increase socio-economic development in the area. It includes factors such as recreational space, educational opportunities, and community cohesion.

What importance do you place on improving livelihood conditions of involved people and local citizens in the area?

Put Weight here (0-10):
Give an explanation of the given weight:

6. Human well-being

This category relates to the enhancement of human well-being benefit in the area from implementing NBS. The importance of this category reflects on health and well-being such as mental well-being, physical activities/health, urban heat island effect mitigation, air pollution improvement through capture/removal by vegetation etc.

What importance do you place on improving human well-being of involved people and local citizens in the area?

Put Weight here (0-10):
Give an explanation of the given weight:

Sub-goals

1.1 Flood risk reduction in urban areas and around rivers, lakes, watercourses, etc.

This sub-goal considers the importance of flood risk reduction in the area. This includes urban areas, rivers, lakes and watercourses. It also means to reduce flood vulnerabilities, reduce flood hazards, reduce flood peaks, slow down runoffs and delay flood peaks.

What importance do you place on reducing the flood risk in the area?

Put Weight here (0-10):
Give an explanation of the given weight:

2.1 Improve water quality in rivers/watercourses, lakes/ponds

This sub-goal indicates whether the improvement of water quality in surface water bodies like rivers and lakes is required. Change in water pollution (i.e., both point sauces and non-point sources), heavy metals, nutrient contamination and sediment deposition, are important key indicators, which can lead towards problems for ecological and human health.

What importance do you place on improving the water quality in surface waters in the area?

Put Weight here (0-10):
Give an explanation of the given weight:

2.2 Improve coastal water quality

This sub-goal reflects on the water quality in coastal areas. The main key factor here is the pollution level of coastal waters. Most ocean pollution is influenced by the land, either along the coastline or coming from the. It causes dangerous conditions for marine life and human health.

What importance do you place on water quality of coastal waters in the area?

Put Weight here (0-10):
Give an explanation of the given weight:

2.3 Improve groundwater quality

Groundwater is a very important source in terms of drinking water supply in many nations. Groundwater quality is less prone to contamination than surface water but can also be affected by various pollution sources and infiltration through the soil. The indicators can be attenuation of pollution in groundwater, change in soil quality or seawater intrusion.

What importance do you place on improving the groundwater quality in the area?

Put Weight here (0-10):
Give an explanation of the given weight:

3.1 Increase habitat area (quantity)

This sub-goal focuses on the importance of increasing the habitat area (i.e., change in riparian, aquatic, wetland and terrestrial habitats for local species). The size of the habitat has significant effects on reproduction and population persistence for various species. Increasing green space can improve population growth and reduce the risk of species extinction.

What importance do you place on increasing room for habitat in the area?

Put Weight here (0-10):

Give an explanation of the given weight:

3.2 Habitat provision and distribution (quality)

Habitat provision and distribution refers to the quality state of a habitat. This can be seen in the distribution of green space in the area and whether habitat structures are showing good connectivity with each other. Better connectivity of habitat structures leads to a higher ecosystem function where higher diversity of animal and plant species can be found, leading to better overall ecosystem health.

What importance do you place on improving the quality of habitat in the area, such as habitat connectivity?

Put Weight here (0-10):

Give an explanation of the given weight:

3.3 To reflect the ecological status and physical structure of habitats

This sub-goal indicates the significance of carrying out conservation and protection strategies to reflect on the ecological status and physical habitat structure of the area. This can be done by monitoring changes of vegetation, erosion protection and the overall conservation status and by looking at possible trends and future projections.

What importance do you place on reflecting the ecological status and physical habitat structure in the area?

Put Weight here (0-10):

Give an explanation of the given weight:

3.4 Land use type

This sub-goal relates to the type of land that could potentially be used or changed to implement a possible NBS. A low impact space (e.g. rooftop) can be used to increase its values of ecosystem services and functions. Often compromises are needed to prevent conflicts in those areas and provide sustainable solutions.

What importance do you place on choosing the right land-use type for possible implementation in the area?

Put Weight here (0-10):

Give an explanation of the given weight:

4.1 To maintain and enhance biodiversity

This sub-goal relates to the importance of maintaining and enhancing biodiversity, which means improving number and types of protected species, native species and their density and diversity in general.

What importance do you place on the maintenance and enhancement of biodiversity in the area?

Put Weight here (0-10):

Give an explanation of the given weight:

4.2 Reduce disturbance to ecosystems

Reducing the disturbance to the ecosystem, in this case, means to decrease stressing ecological factors in the area such as specific amounts of non-native/migrated and invasive species that could be a threat to the local ecosystem or local species.

What importance do you place on reducing the disturbance to ecosystems in the area?

Put Weight here (0-10):

Give an explanation of the given weight:

5.1 Increase recreational opportunities

Increased recreation opportunities mean providing recreational space, giving the possibility to people to spend their free time in the area, engaging livelihood activities and increasing number of tourists. These can be beneficial for human well-being from both biological and psychological aspects.

What importance do you place on increasing recreational opportunities in the area?

Put Weight here (0-10):

Give an explanation of the given weight:

5.2 Education and awareness

A site can be seen as an opportunity to provide knowledge and awareness about connected key aspects such as the reduction of major risks (e.g. flooding) in the area as well as supporting ecosystems.

What importance do you place on increasing or improving educational/awareness opportunities through NBS in the area?

Put Weight here (0-10):

Give an explanation of the given weight:

5.3 Maintain and if possible enhance cultural values

Maintaining and (if possible) enhancing cultural values present in the area includes reducing the risk of losing cultural heritage during extreme weather events. Also, it means to improve working environments for local food production and to maintain or increase numbers of cultural events.

What importance do you place on maintaining or enhancing cultural values in the area?

Put Weight here (0-10):

Give an explanation of the given weight:

5.4 Accessibility

Accessibility reflects on the factor of how easy it is to reach/ access the NBS site. It includes free space of the area and connection between area and surrounded homes.

What importance do you place on the accessibility to an NBS in the area?

Put Weight here (0-10):

Give an explanation of the given weight:

5.6 Improve Community Cohesion

Improve community cohesion is enhanced when the people are engaged in an activity which connects them. This also includes the number of people who communicate with the neighbourhood in the NBS area and cognitive and social development in children and young people.

What importance do you place on improving community cohesion in the area?

Put Weight here (0-10):

Give an explanation of the given weight:

5.7 Stimulate/increase economic benefits

This sub-goal shows the significance of increasing or stimulating economic growth in the area. This includes factors such as avoided future damage costs and reduced costs from energy and carbon saving through NBS (e.g. reduced energy consumption of buildings). Also, factors can be seen in reducing the costs of negative health impacts and an increase in property values.

What importance do you place on stimulating/increasing economic benefits in the area?

Put Weight here (0-10):

Give an explanation of the given weight:

5.8 Encourage new business models and other community benefits provided by NBS

This sub-goal refers to the opportunity of encouraging new business models and community benefits to arrive through a NBS. Due to increasing attractiveness of the area, new businesses could be interested in settling, also through the improvement of living and working conditions. Furthermore, this could mean an increase in created green jobs in the area.

What importance do you place on encouraging new business opportunities and benefits to the community in the area?

Put Weight here (0-10):

Give an explanation of the given weight:

6.1 Direct health and well-being impacts

Direct health benefits can be considered in terms of mental and physical well-being. One of the main reasons why an NBS could improve human health can be seen in the fact that it allows increasing physical activity to the people who live or visit the area, spending more time outside and being more engaged within the community.

What importance do you place on improving direct health impacts in the area?

Put Weight here (0-10):

Give an explanation of the given weight:

6.2 Indirect health and well-being impacts

Indirect health impact can be seen due to improving air quality (e.g. removing pollution with planted vegetation), less noise pollution, possible reduction of heavy metal contents in the environment and the mitigation to the urban heat island effect. NBS can reduce the exposure level of toxic substances towards humans and can be beneficial in terms of human health.

What importance do you place on indirect health impacts in the area?

Put Weight here (0-10):

Give an explanation of the given weight:

Supplementary 3.3 Questionnaire for collecting stakeholders' preferences on applicable/suitability of measures

An explanation

This section provides some information about NBS measures. Definition, primary function and co-benefits are given for each NBS measure, with a picture that should give a better understanding of how the measure would look like in reality.

Weights should be given with **values from 0 to 10** in order to show the suitability to address the present issues and developments in the area. A value of **0** represents **inapplicable/unsuitability**, whereas **10** means that the measure is **very suitable**.

1. Floodplain excavation/enlargement/restoration

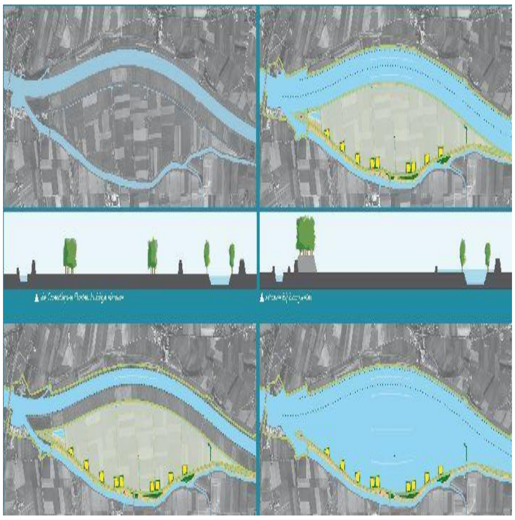

A floodplain is an area bordering a river that naturally provides space for the retention of floods and rainwater. Floodplains have often been drained, and in many places, they have been separated from the river by structures.

The floodplain can be enlarged by lowering the level or/and increasing the width of the floodplain area. An area of the floodplain increases will create more room for the river during high flow by increasing the discharge capacity and provide upstream retention.

Sediments can also cover the floodplains. By allowing the natural functioning of rivers, floodplain restoration measures have high potential to control runoff and reduce flood risk, since they should aim to maximise the capacity of the floodplain to store river water. The associated changes in land use and reduction in surface-runoff can lead to higher recharge of water into the ground. Increased organic matter content can increase soil water retention, while removal of sediment improves soil permeability.

Co-Benefit: The floodplain excavation creates opportunities for nature and recreational development. With a significant change of land cover, it can reduce pollution by activating filtration by vegetation and soil. Floodplain restoration enables recovery of natural erosion and sedimentation processes, therefore reducing sediment transport downstream.
Floodplain contributes to creating terrestrial, aquatic and riparian habitats, increasing fish populations, improving biodiversity and providing natural biomass. The restoration site can be planted with native grasses, shrubs, and trees, which will discourage the establishment of invasive vegetation.
Floodplains are likely to contribute to climate change adaptation through the fixation of carbon dioxide by photosynthesis and C-burial. They also provide recreational opportunities and aesthetic value.

Put Weight here (0-10):

Give an explanation of the given weight:

2. Depoldering

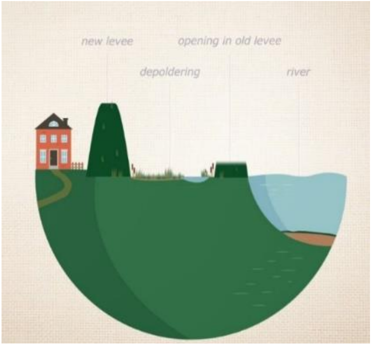

The dike on the riverside of a polder is relocated land-inwards, which return the polder to the river. This way, the river has more room for flooding due to the water can flow into the area at high water levels.

A new dike is built further inland. Breaches are created in the old dikes, allowing the tides to flow in and out of the area. This is how tidal nature, with mudflats and marshes, develops in the depoldered area. At the same time, the force that the water exerts on the dikes is tempered, reducing the likelihood of floods further inland.

Co-Benefit: Depoldering creates opportunities for nature habitat developments as well as improve aesthetic value in the area. It also offers opportunities for recreation activities and economic activity.

Put Weight here (0-10):

Give an explanation of the given weight:

3. Widening of water bodies

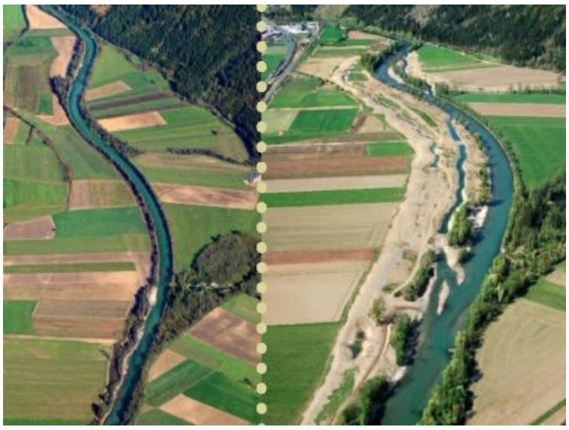

The river width is an activity which aims to increase the conveyances characteristics of the river though widening by excavating the embankment. The widening rivers provide more space in the river. By enlarging the cross-sectional area, it increases the bank discharge of the river along with its hydraulic radius. This will increase the velocity of the river and reduce the chances of it flooding in the immediate area by moving the floodwater further on downstream.

Co-Benefit: Channel migration and braiding are enabled within the widened reach, leading to greater structural, hydraulic, and habitat heterogeneity. The exchange between the water column and channel bed and river banks (vertical and lateral linkages) can affect water quality because these are biogeochemically active areas where organic matter decomposition and nitrogen removal occurs.

Put Weight here (0-10):
Give an explanation of the given weight:

4. Deepening water bodies

The water body bed is deepened by excavating the surface layer of bed to increase the depth of water bodies such as rivers, canals or ponds. The deepened water body bed provides more room for the water.

Increasing the depth of water bodies is also increasing the storage volume. The amount of water which can be stored in this way can become available when water is scarce. The water bodies are refilled when water is abundant during wet periods. Deepened water bodies help to reduce flood risk as rivers can transport a larger amount of water to downstream and ponds and lakes have a larger retention capacity.

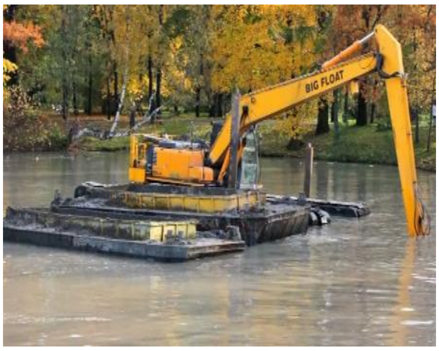

Co-Benefit: Deepening water bodies contributes to improving the status of Physico-chemical and hydro-morphology quality elements. Diversifying water depth contributes to improving the diversity of habitats offered by the river and to create new habitats. Deepening water body also fosters the development of riparian habitats on river banks. This leads to enhanced natural biomass production and helps to create and preserve biodiversity. The measure also contributes to better management of fish stocks and helps to improve the status of biology quality elements and prevent surface water deterioration. Deepening water bodies can provide recreational opportunities (through the diversification of the activities offered by the river) and aesthetic value.

Put Weight here (0-10):
Give an explanation of the given weight:

5. Lowering groynes

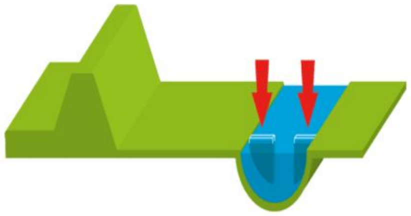

Groynes stabilise the location of the river and ensure that the river remains at the correct depth. However, at high water levels groynes can form an obstruction to the flow of water in the river. Lowering groynes increases the flow rate of the water in the river.

. A perpendicular groyne is constructed at a right angle to the flow of the river. These groynes will be lowered or removed. By lowering the groynes in the river and building parallel barriers, the river will be able to drain excess water easier

Co-Benefit: Lowering the groynes are more navigable depth for shipping and wet banks at low water levels during dry periods. In the side stream of lowering groynes, development of flora and fauna is expected to improve along the river banks.

Put Weight here (0-10):
Give an explanation of the given weight:

6. Bypass/diversion channels

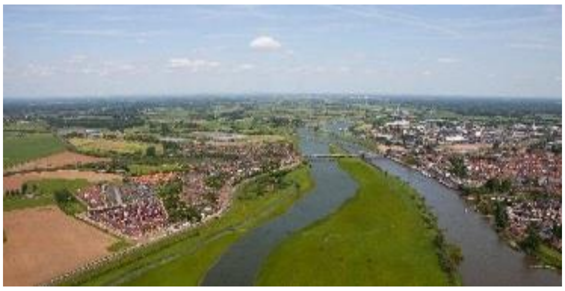

Bypass channels divert river flows at a point upstream of an area. These diverted flows can be discharged back to the same river (Bypass channel), or into another natural drainage system nearby (Diversion channel). Gates usually use to regulate flows into bypass and diversion channels. The measure implies 'giving back' floodplain area to the river. It is a separate channel into which floodwaters are directed to lessen the impact of flooding on the main river system.

Co-Benefit: Apart from enhancing the rivers' capacity, many secondary benefits are produced such as increasing groundwater infiltration, improving water quality, restoring natural floodplain forming processes (e.g., sediment transport and deposition) and improving fish and wildlife habitats. (FEMA, Floodplain Natural Resources and Functions, accessed on Sept. 2018)

With reduced flows in the main stem of the river, streamside vegetation can encroach into the river channel, thereby changing its physical and biological characteristics and restoring the natural floodplain ecosystem. The in-stream habitat component is providing good elements of abundant cover, clean substrates and high base flows, which assures a stable water supply with adequate depths and flow during droughts.

Recreational opportunities can best be enhanced by developing additional access facilities on the diversion channel to relieve current crowded conditions.

Put Weight here (0-10):

Give an explanation of the given weight:

7. Reconnection of oxbow lakes and Re-meandering

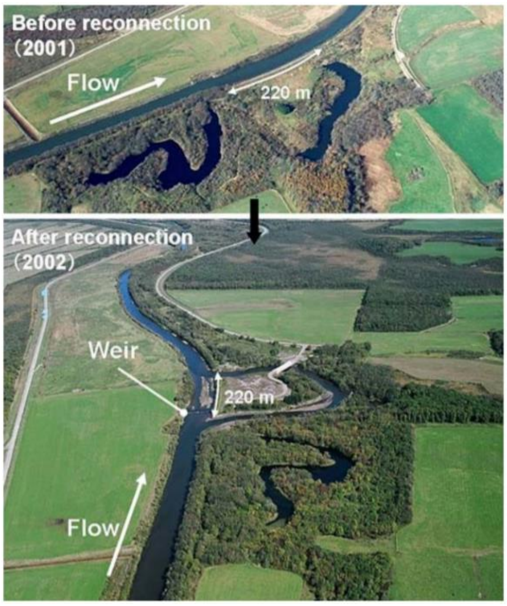

Oxbow lakes are former meanders that have been cut off from the river, thus creating a small lake with a U form. Oxbow lakes occur naturally, but may also occur due to artificial river straightening. Reconnecting an oxbow lake/re-meandering involves removing terrestrial lands between both water bodies, therefore favouring the overall functioning of the river by restoring lateral connectivity, diversifying flows and cleaning the river section of the present oxbow, and thereby providing better water retention during floods.

Co-Benefit: Oxbow lakes and reconnected side arms may play an important role in creating habitats, but care should be paid not to destroy pre-existing oxbow lake habitats. Often these habitats are used for spawning places by fish and other aquatic groups so that fish stocks can increase. That, in turn, contributes to improving the status of biology quality elements.

Bank vegetation often expands after reconnection because of improved water regime, and populations of water birds, amphibians, reptilian and mammal species can increase.

Restoration of natural green areas significantly contributes to the 2020 Biodiversity Strategy and provides aesthetic and cultural value.

Put Weight here (0-10):

Give explanation of the given weight:

8. Retention ponds

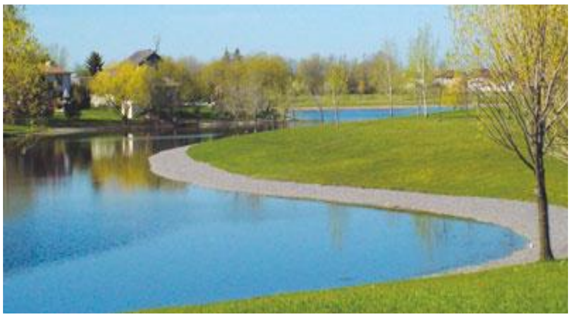

Retention ponds are ponds designed with additional storage capacity to attenuate surface runoff during rainfall events. Ponds are created by using an existing natural depression, by excavating a new depression, or by constructing embankments. Increasing storage can be applied on different scales.

Retention ponds reduce peak runoff through storage and controlled outflow and reduce the risk of surface flooding in conjunction. Reduction of discharge causes lower water levels downstream of the site of the measures.

Co-Benefit: Retention basin also help to prevent downstream erosion, and improve water quality in an adjacent river, stream, lake or bay thought vegetation. Sometimes they act as a replacement for the natural absorption of a forest, or other natural proves that was lost when an area is developed.

Retention ponds can be effective at pollutant removal. They are also highly effective at intercepting sediment. Through reducing diffuse pollution, retention ponds play a role in preserving and improving surface water quality.

Creation of ponds will create new aquatic and riparian habitat, therefore increasing natural biomass production and contributing to biodiversity preservation.

Retention ponds can contribute to more sustainable agricultural practices. Ponds also increase the aesthetic/cultural value of the landscape.

Put Weight here (0-10):
Give an explanation of the given weight:

9. Detention basins

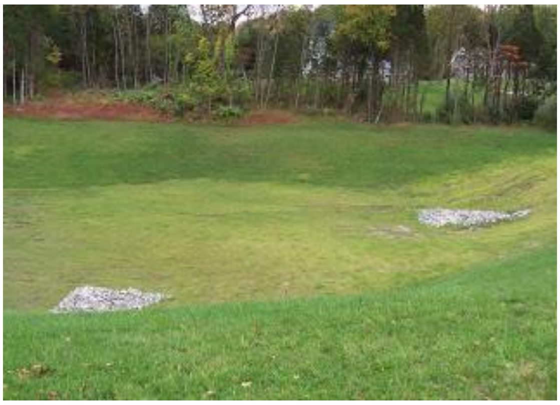

A detention basin is a free space from water in dry weather. It designs to store runoff for temporary during high flow then releasing it slowly to downstream or a nearby watercourse, using an outlet control structure to control the flow rate.

Detention basins are vegetated depressions designed to hold runoff from impermeable surfaces and allow the settling of sediments and associated pollutants. They are not designed to allow infiltration. The storage capacity is dependent on the design of the basin, which can be sized to accommodate any size of the rainfall event. Detention basins can reduce the risk of surface flooding in conjunction with other NBS features, and in doing so, contribute to climate change adaptation.

Co-Benefit: Detention ponds and basins can be effective at capturing sediment in urban or rural runoff and at pollutant removal, as a result of the settling of particulate pollutants and uptake by vegetation. Therefore, they have the potential to improve water quality in receiving water bodies by addressing diffuse urban pollution and reducing chemical pollution.

Detention basins may provide minor biodiversity benefits (although unlikely to provide significant habitat improvements). Where used to intercept and store runoff from low permeability surfaces in agricultural areas, detention basins can contribute to more sustainable agricultural practices. By creating green areas, they provide aesthetic and recreational benefits.

Put Weight here (0-10):
Give an explanation of the given weight:

10. Dike removal/relocation

Relocating a dike land-inwards increase the width of the floodplains and provide more room for the river. This entails exposing land that once had been protected by the dyke to high water in order to expand the river's winter bed. Relocating dikes increase conveyance in the floodway by enabling floodwaters to spread out and slow down. This reduces the height of floodwaters, reduces peak flows in receiving watercourses, and consequently reduce flood risk and erosion. Moreover, it reduces the pressure on the dike itself.

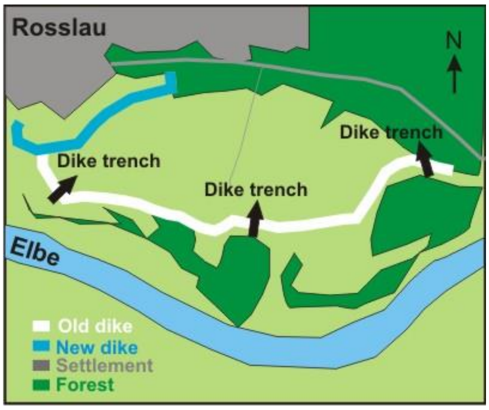

Co-benefit: Dike relocation creates new retention area, which can give more room for the river by opening former floodplains. This will also benefit in improving dynamics and functions of ecosystem and biodiversity. Reduced flows also contribute to the filtration of pollutants, potentially improving surface water qualitative status and preventing surface and groundwater status deterioration. Moreover, this will enhance in creating recreational opportunities. If the new construction of dike includes hiking or biking, this will be able to provide both a recreation opportunity for neighbouring communities and accessibility to the restored floodplain and riparian habitats.

Put Weight here (0-10):

Give an explanation of the given weight:

11. Removing obstacles

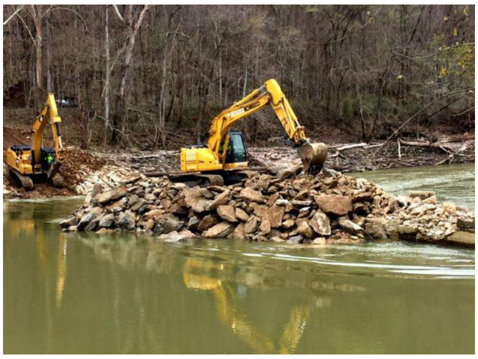

Removing or modifying obstacles, such as bridge, abutments, factories on mounds, involves the removal of man-made elevations which disturb the free flow of the river. Removing or modifying obstacles can increase the flow rate of the water in the river and reduce the water levels upstream of the measure.

The removal of hydraulic obstacles in the floodplain is another way to increase its discharge capacity without increasing its water level.

Co-Benefit: Removing obstacles add sheer surface area to the floodplain and reduce the visible remains of human activity (artefacts). This enhances the naturalness of the area and the diversity of natural habitats.

Put Weight here (0-10):

Give explanation of the given weight:

12. Wetland restoration/enhancement

A wetland is an area of marsh, fen, peatland or water for whether temporary or permanent. Wetlands restoration can store water and slowly release water. Wetlands often dense vegetation and are usually flat area, which contributes to slowing runoff by reducing peak flow and reduce stream discharge. Therefore, it reduces flood risks as well as in coastal areas can support protection against seat storms and storm surges.

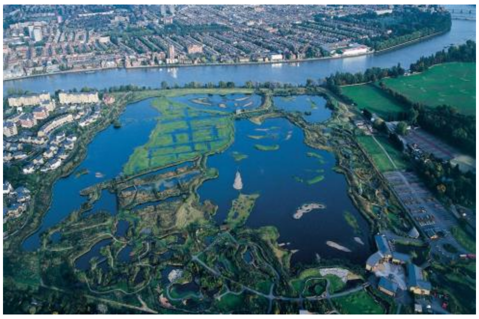

Co-benefits: Wetland provides a wide range of socio-economic co-benefits. Wetlands contribute to water quality through their natural ability to filter effluents and absorb pollutants. Microorganisms in the sediment and vegetation in the soil help to break down many types of waste, eliminate pathogens and reduce the level of nutrients and pollution in the water

They create aquatic and riparian habitat. It is potentially providing large areas of natural habitat that is valuable for recreation activities, livelihood opportunities and education opportunities.

Put Weight here (0-10):

Give an explanation of the given weight:

13. Lake restoration

A lake is a natural water retention facility. In passing through the lake, river water is not only slowed down but also has its physics-chemical characteristics altered/ regulated. These mechanisms contribute to reducing peak flows in receiving watercourses, effectively maintaining the natural flood risk management capacity of a catchment. Protection against floods can be improved through an integrated strategy taking into account all water uses.

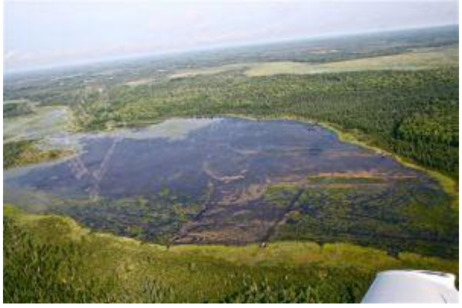

Co-benefits: A lake can store water (for flood control) and provide water for many purposes such as water supply, irrigation, fisheries, tourism, etc. Besides, it serves as a sink for carbon storage and provides important habitats for numerous species of plants and animals, including waders.

Lake restoration has the potential to improve water quality in receiving water bodies. It also preserves aquatic habitats and can increase species diversity. Along with the benefits to temperature and water quality, this can contribute to increase fish stocks. Restoration of the lake and their surroundings can also benefit riparian vegetation and species, and provide an overall increase in biomass production. As a result of improving the production of phytoplankton and zooplankton, creates optimum conditions for aquatic and terrestrial ecosystems.

Lake restoration is thus a key measure for reaching good water ecological status. Lake can have recreational and cultural benefits, becoming popular areas to visit, for example for sailing, fishing and bird watching.

Put Weight here (0-10):
Give an explanation of the given weight:

14. Buffer strips

Riparian forest buffers can have more significant in holding water than cutover or non-forest covered areas. Riparian buffers serve to slow water as it moves off the land. Because of their rougher ground surface, they can slow runoff more effectively than bare ground. However, riparian forest buffers have a limited ability to store and slow terrestrial runoff due to their relatively small breadth.

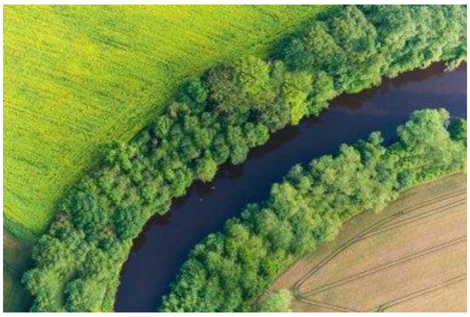

Co-benefits: By preserving a relatively undisturbed area adjacent to open water, riparian buffers can serve several functions related to water quality and flow moderation. The trees in riparian areas can efficiently take up excess nutrients and may also serve to increase infiltration. Forest riparian buffers can play an important role in biodiversity preservation, both by direct provision of riparian habitat and by providing habitat "corridors". They contribute to the creation of aquatic habitat by moderating the stream temperature regime and by acting as a source of coarse woody debris. Riparian buffers also increase the recreational value of the area.

Put Weight here (0-10):
Give an explanation of the given weight:

15. Afforestation/Forests and naturally vegetated land

Afforestation is the process of planting trees, or sowing seeds, in areas that have ever been forested to create a forest. Forests can help to reduce public costs for stormwater management, flood control, transportation, and other forms of built infrastructure. Trees (as well as other vegetation covers in the catchment), intercept rainfall and increase infiltration thus moderating both the runoff into the river system and the storage of water in the soil. The ability of soils in forest areas to store water and release it through seepage, transpiration and evaporation helps in regulating the water supply in the catchment. Forests and areas with good vegetation cover can moderate extreme events by reducing the likelihood (or frequency) of floods, landslides, mudflows and avalanches, which can cause extensive damage to infrastructure and inhabited areas.

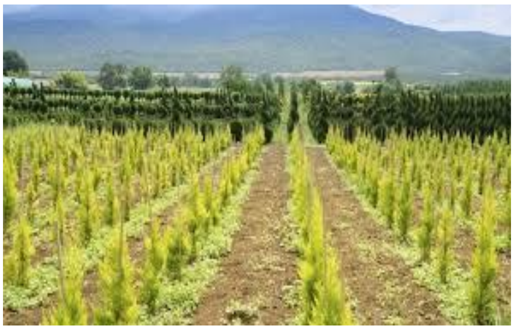

Co-benefits: Afforestation play a pretty important role when it comes to decreasing the greenhouse effects. We all know that greenhouse gases represent a contributing factor that triggers climate change all around the globe. Forests can also protect biological diversity and preserve essential ecological functions while serving as a place for recreation and civic engagement.

Some afforestation might be developed by companies to grow trees and improve the production of charcoal and timber. Furthermore, afforestation also constitutes more job opportunities. It also helps local businesses to increase the supply of its products while also benefiting the local economy.

Put Weight here (0-10):
Give an explanation of the given weight:

16. Reforestation and forest conservation

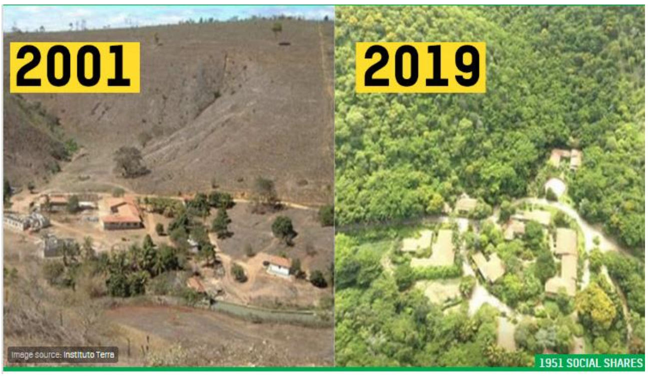

Reforestation is the restoration of forests in areas where forests were removed or destroyed. Forest growth prevents water runoff. That is because the forested area will release its water to the mainstream later than water running off pastureland. The water then seeps into the soil and recharges groundwater. This will increase the water supply for both animals and people. Trees and other plants also enrich the soil and increase its quality, they prevent the process of soil erosion and landslide, also provide better water-retention.

Co-benefits: Reforestation can be used for both as mitigation and adaptation measures for climate change. It can cause dramatic and rapid accumulation of carbon in tree biomass as trees can capture and store carbon dioxide from the atmosphere during photosynthesis, and convert it into oxygen and carbohydrates. Thus, this helps mitigate global warming by reducing the growth of carbon emissions to the atmosphere. Reforestation can also be used to improve the quality of air by removing air pollutants such as sulphur dioxide, ammonia and nitrogen dioxide from the air. The tree roots also prevent erosion that can wash away good soil. Trees also help to rebuild ecosystems and habitats as trees provide homes for animals and birds.

Put Weight here (0-10):
Give an explanation of the given weight:

17. Upper watershed restoration

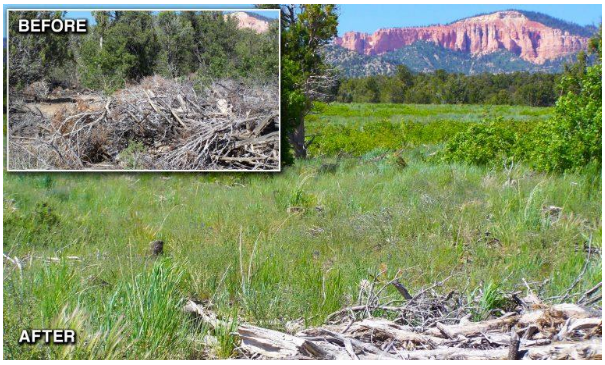

Upper watershed restoration aims to improve infiltration; thus, precipitation can infiltrate to the ground without flowing as runoff. Watershed restoration often uses bio-engineering techniques. Bio-engineering combines living plants with dead plants or non-living material to produce a living system that resists erosion. Selective planting on a hillside is an example of bioengineering. When properly completed, the bioengineered solution can produce an environment that stabilises the land, controls flooding and even takes part in the treatment of water.

Co-benefits: Restoration of a watershed can help the ecosystem to return as close as possible of original. The restorative process includes the remediation of the water quality, repairing the source of the water damage, and repopulating the watershed with plant and animal species. In some cases, it is sufficient to make the restored habitat attractive to native species and to allow natural repopulation. Restoring watersheds is important to species health, which is part of the ecosystem and to the communities that depend on the watershed for their drinking water.

Put Weight here (0-10):
Give an explanation of the given weight:

18. Natural bank stabilisation

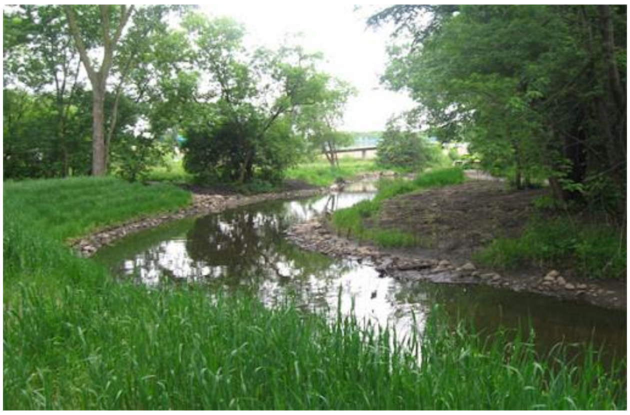

Natural bank stabilisation involves recovering its ecological components, thus reversing such damages and allowing the bank to be stabilised, as well as allowing the river to move more freely. Riverbank renaturation consists in recovering its ecological components, thus reversing such damages and especially allowing the bank to be stabilised, as well as rivers to move more freely. Replacing concrete banks with natural vegetation also generally increases the roughness of the bank and hence slows down river flow. Thereby, this measure can contribute to reducing flood risk.

Common techniques of natural bank stabilisation are bank-re-vegetation and bio-engineering. Bank re-vegetation is very effective in mitigating the erosive effects of overland flood flows eroding down the banks as waters recede. Bio-engineering techniques for bank stabilisation incorporate the use of riparian vegetation and wood cuttings that are installed in the bank to provide structural stability. Bio-engineering techniques are used to enhance slope stability, control sediment generation, and maintain bio-diversity.

Co-Benefit: An increased surface area of natural materials allows for increased natural filtration and biological pollutant decomposition, which contributes to increasing the capacity of the river to purify the water naturally. Stabilising banks prevents river flow from eroding the river banks, although activating the typical hydro-morphological processes can lead to small scale erosion and sedimentation and the development of a broad and gently sloping bank profile.

By slowing down the flow and giving back its natural features to the river, natural bank stabilisation creates aquatic and riparian habitats, thus potentially increasing fish populations and natural biomass production, improving the status of biology quality elements and preserving biodiversity. Replacing concrete banks with natural materials and vegetation also improves the aesthetic value of the area.

Put Weight here (0-10):
Give an explanation of the given weight:
